# Supplementary material for: Acceptability of a behavioural intervention to mitigate the psychological impacts of COVID-19 restrictions in older people with long-term conditions: a qualitative study
Source: BMJ Open. 2023 Mar 13;13(3):e064694. doi: 10.1136/bmjopen-2022-064694 (PMC10015671; doi:10.1136/bmjopen-2022-064694)
Supplement: Supplementary data [file bmjopen-2022-064694supp001.pdf]

Appendix 1 Themes from thematic analysis (TA)

Theme	Descriptive summary of theme	Illustrative data extracts
Intervention context	The COVID-19 restrictions applied variably to older adults in the BASIL study (e.g., shielding, social distancing) dependent on their health status. There was considerable variability in how the restrictions had impacted participants' mental health.	*I feel as though I'm held in, I'm being persecuted because I was shielding originally. I did everything that was asked of me, we're now moving out but all of a sudden because of political decisions, I'm now finding myself back where I was. Staying in a lot, don't meet a lot of people at the best of times, generally speaking you'll probably say loneliness, isolation. (OA06)*<br><br>*But I just didn't feel motivated to do anything 'cause all the things that I really had been enjoying had just suddenly stopped overnight, literally*. (OA14)<br><br>*It's just a worry, obviously I don't want to catch it, you know, because of my underlying conditions I think I would have a rough time of it, you know …So, I'm quite happy to isolate myself, if you like (OA12)*<br><br>*The constant changes in rules and the confusion that people have had over that, has, you know…it has affected it [intervention delivery]. Because one week, someone can be doing okay and the next week, rules have changed and they're feeling anxious, so they're not really sure where they stand anymore. Or, you know, restrictions have been lifted. And so one week, they're really struggling and the next week they've been able to*

		go back to doing most of what they normally do, and actually, they're loads better. So it changes week to week. So you really don't know from one week to the next, where someone's going to be at, dependent on, you know, the restrictions in their area. (BSW07)
	Remote consulting / perceived lack of access to primary care	*My diabetes, I think, they've forgot about me, I have no idea I haven't…the last two appointments were supposed to be phone ones and they didn't mat…they were cancelled for different reasons. My cancer ones, I don't know what's happening there, I've missed the last two because of the same problem. They've rang up to say that either the person's not available or the computer's broke down, or…so that's my cancer and my diabetes. (OA11)*<br><br>*There's been these two sides of things. There's one side, it's not affected their long-term conditions or their access in the support. The other side is where they're not wanting to access the support because they're either too scared or they don't want to go out, and then that's having the impact on their long-term conditions 'cause they're not being able to go to their routine appointments for it and things like that. (BSW06)*
Study entry	Phased approach to recruitment (i.e.: 1) initial text from GP surgery, 2) letter with study participant information (sent via GP surgery), 3) phone call from the Clinical Research	*So, I was really quite down in the dumps a bit. And then I got this text from my doctor saying that I should take part in…[they] thought it would be beneficial for me to take part. So, I said yes, I would do that and I rang the number and said yes I would.* (OA01)

	Network and 4) phone call from the University study team), was both feasible and acceptable. It seemed to provide successively layered opportunities for participants to gain sufficient information and decide if they wanted to participate in the BASIL study.<br><br>Need for targeted recruitment.	*No, I mean the doctor gives you a warning that this is about to happen and then you're prepared when somebody phones up that it's not a scam, a con, which is what I don't worry about it because I know how to deal with it but to some people it could be worrying. (OANC17)*<br><br>*I think it's maybe thinking about who we would want to target in the main study. I think as I say, the participants I was working with didn't consider themselves to be struggling with their mood, they'd come into the study for altruistic reasons, rather than looking to boost their own mood and wellbeing. I guess maybe to be more targeted. (BSW04)*
Intervention delivery and content	The BASIL pilot intervention was solely delivered by telephone to older adults in their homes. Online intervention delivery was offered; however, this option was not taken up by any participants.<br><br>Duration of intervention	*I: receiving the support package over the telephone, how was that?*<br>*P: Well, I found that the person I was speaking with showed a lot of empathy, understood where I was coming from, you know, just about being on your own for long periods of time and that sort of thing. (OA06)*<br><br>*And I thought, I don't want to be bothered with not being able to get it [online platform] on, you know, I'd rather a telephone call and then I don't have the worry of the [online platform] going wrong. (OA15)*<br><br>*So if they know they've got a telephone call on a*

		Wednesday morning at 11, then that will really help them to structure their week and give them something to look forward to and mark their weeks. (BSW05)<br><br>*So for those who have more severe low mood, when I start working with them, I definitely see that they have more to learn in terms of using technology. (BSW06)*
	Self-help booklet	*I think it was enough because we'd done the whole book and it left me, at the end, to have an action plan. So that's all there, I'd written out on the page, so I've got that to refer to. Yes, I think eight weeks was enough. (OA15)*
	BA	*My first thought was, it was in very simple, precise language so that anybody could understand it. It wasn't, you know, sort of above anybody's head if you know what I mean. I thought it was very good, very simple. And it had different examples of what people were doing and how they were overcoming their difficulties. Yes, I thought it was very good, I liked it. (OA15)*<br><br>*And there was also plenty of opportunity for me to put my two pennorth worth in, you had pages that were blank and…list your three most priority items today……. You know, that was so…so it gave you that. (OA06)*

		with the booklet and the [person] who I was talking with, we were able to say what is it about you that's so important that you must have this priority and that priority, and they compete, why don't you break it down. What can I do, how much can I do, how does it interwork with each other, and it's that breaking down of… catastrophic how you are, obviously, the pandemic, how does the pandemic impact you, what can you do to circumvent it, you know, that sort of thing. And that was very useful. (OA06)<br><br>*Also, probably the breaking things down into making them more manageable. That's been particularly useful for physical health. So that helped with the physical pain. (BSW06)*<br><br>*And I felt really good to tell {them] that it made me feel better, it made me feel good. The support I got from [them] was really good because things that I'd never thought about, [they] said [themselves], why don't you…you know, have you tried this? (OA01)*
