# Supplementary material for: Acceptability of a behavioural intervention to mitigate the psychological impacts of COVID-19 restrictions in older people with long-term conditions: a qualitative study
Source: BMJ Open. 2023 Mar 13;13(3):e064694. doi: 10.1136/bmjopen-2022-064694 (PMC10015671; doi:10.1136/bmjopen-2022-064694)
Supplement: Author's manuscript [file bmjopen-2022-064694.draft_revisions.pdf]

BMJ Open

BMJ Open is committed to open peer review. As part of this commitment we make the peer review history of every article we publish publicly available.

When an article is published we post the peer reviewers' comments and the authors' responses online. We also post the versions of the paper that were used during peer review. These are the versions that the peer review comments apply to.

The versions of the paper that follow are the versions that were submitted during the peer review process. They are not the versions of record or the final published versions. They should not be cited or distributed as the published version of this manuscript.

BMJ Open is an open access journal and the full, final, typeset and author-corrected version of record of the manuscript is available on our site with no access controls, subscription charges or pay-per-view fees (http://bmjopen.bmj.com).

If you have any questions on BMJ Open's open peer review process please email info.bmjopen@bmj.com

BMJ Open

Acceptability of a behavioural intervention to mitigate the psychological impacts of COVID-19 restrictions in older people with long-term conditions: a qualitative study

Journal:	*BMJ Open*
Manuscript ID	bmjopen-2022-064694
Article Type:	Original research
Date Submitted by the Author:	27-May-2022
Complete List of Authors:	Shearsmith, Leanne ; University of Leeds, Leeds Institute of Health Sciences<br>Coventry, Peter; University of York, Department of Health Sciences; University of York, York Environmental Sustainability Institute<br>Sloan, Claire; University of York, Department of Health Sciences<br>Henry, Andrew; University of York, Department of Health Sciences; Tees Esk and Wear Valleys NHS Foundation Trust, Research & Development<br>Newbronner, Liz; University of York<br>Littlewood, Elizabeth; University of York, Health Sciences<br>Bailey, Della; University of York, Health Sciences<br>Gascoyne, Samantha; University of York, Department of Health Sciences<br>Burke, Lauren; University of York, Department of Health Sciences<br>Ryde, Eloise; University of York, Department of Health Sciences; Tees Esk and Wear Valleys NHS Foundation Trust, Research & Development<br>Woodhouse, Rebecca; University of York<br>McMillan, Dean; University of York, Department of Health Sciences; Hull York Medical School<br>Ekers, David; University of York, Health Sciences; Tees Esk and Wear Valleys NHS Foundation Trust, Research & Development<br>Gilbody, Simon; University of York, Department of Health Sciences<br>Chew-Graham, Carolyn; Keele University, School of Medicine
Keywords:	COVID-19, Depression & mood disorders < PSYCHIATRY, QUALITATIVE RESEARCH

SCHOLARONE™
Manuscripts

BMJ

I, the Submitting Author has the right to grant and does grant on behalf of all authors of the Work (as defined in the below author licence), an exclusive licence and/or a non-exclusive licence for contributions from authors who are: i) UK Crown employees; ii) where BMJ has agreed a CC-BY licence shall apply, and/or iii) in accordance with the terms applicable for US Federal Government officers or employees acting as part of their official duties; on a worldwide, perpetual, irrevocable, royalty-free basis to BMJ Publishing Group Ltd ("BMJ") its licensees and where the relevant Journal is co-owned by BMJ to the co-owners of the Journal, to publish the Work in this journal and any other BMJ products and to exploit all rights, as set out in our licence.

The Submitting Author accepts and understands that any supply made under these terms is made by BMJ to the Submitting Author unless you are acting as an employee on behalf of your employer or a postgraduate student of an affiliated institution which is paying any applicable article publishing charge ("APC") for Open Access articles. Where the Submitting Author wishes to make the Work available on an Open Access basis (and intends to pay the relevant APC), the terms of reuse of such Open Access shall be governed by a Creative Commons licence – details of these licences and which Creative Commons licence will apply to this Work are set out in our licence referred to above.

Other than as permitted in any relevant BMJ Author's Self Archiving Policies, I confirm this Work has not been accepted for publication elsewhere, is not being considered for publication elsewhere and does not duplicate material already published. I confirm all authors consent to publication of this Work and authorise the granting of this licence.

For peer review only

Acceptability of a behavioural intervention to mitigate the psychological impacts of COVID-19 restrictions in older people with long-term conditions: a qualitative study

Shearsmith L, Coventry PA, Sloan C, Henry A, Newbronner E, Littlewood E, Bailey D, Gascoyne, S., Burke, L., Ryde, E., Woodhouse R, McMillan D, Ekers, D, Gilbody S, Chew-Graham CA.

Corresponding author details:
Leanne Shearsmith, Academic Unit of Primary Care, Leeds Institute of Health Sciences, 10.38b, Level 10 Worsley Building, Clarendon Way, Leeds, LS2 9NL.
Email: l.shearsmith@leeds.ac.uk Phone: 07857 664294 Twitter handle: @lshears_

Authors:

Leanne Shearsmith[a]

Peter A Coventry[b,c]

Claire Sloan[b]

Andrew Henry[b,d]

Elizabeth Newbronner[b]

Elizabeth Littlewood[b]

Della Bailey[b]

Samantha Gascoyne[b]

Lauren Burke[b]

Eloise Ryde[b,d]

Rebecca Woodhouse[b]

Dean McMillan[b,e]

David Ekers[b,d]

Simon Gilbody[b]

Carolyn A Chew-Graham[f]

[a] Leeds Institute of Health Sciences, University of Leeds, Leeds, United Kingdom
[b] Department of Health Sciences, University of York, York, United Kingdom
[c] York Environmental Sustainability Institute, University of York, York, United Kingdom
[d] Tees Esk and Wear Valleys NHS Foundation Trust, Research & Development, Flatts Lane Centre, Middlesbrough, United Kingdom, United Kingdom
[e] Hull York Medical School, Hull, Kingston upon Hull, United Kingdom
[f] School of Medicine, Keele University, Staffordshire, United Kingdom

Keywords: qualitative research, COVID-19, depression, aged, clinical psychology

Word count: 4560 (excluding title page, references, figures and tables)

For peer review only

Abstract

Objectives

The COVID-19 pandemic has heightened the need to address loneliness and social isolation (and associated incidence of depression) amongst older adults. Between June and October 2020, the Behavioural Activation in Social IsoLation (BASIL) pilot study investigated the acceptability and feasibility of a remotely delivered brief psychological intervention (Behavioural Activation, BA) to prevent and reduce loneliness and depression in older people with long term conditions (LTCs) during the COVID-19 pandemic.

Design

An embedded qualitative study was conducted with semi-structured interviews to generate data that was first analysed inductively using thematic analysis and then deductively using the Theoretical Framework of Acceptability (TFA).

Setting

National Health Service and third sector organisations in England.

Participants

Sixteen older adults and 9 Support Workers (BSWs) participating in the BASIL pilot trial.

Results

Older adults and BSWs described a positive affective attitude towards the intervention linked to altruism, however the activity planning aspect of the intervention was limited due to COVID-19 restrictions. The intervention was understood by older adults & BSWs, although less understanding in older adults without low mood. A manageable burden was involved with delivering and participating in the intervention. For ethicality, older adults valued social contact and making changes, BSWs valued being able to observe those changes. Opportunity cost was low for BSWs & older adults. BA was perceived to be useful in the pandemic and likely to achieve its aims, (Perceived Effectiveness) especially if tailored to people with both low mood and LTCs. Self-efficacy developed over time and with experience for both BSWs and older adults.

For peer review only

Conclusions

Overall, the BASIL pilot study processes and BA intervention were found to be acceptable. Use of the TFA provided valuable insights into how the intervention was experienced and how the acceptability of study processes and the BA intervention could be enhanced ahead of the larger definitive trial (BASIL+).

Strengths & Limitations

1. The use of TFA in both informing the topic guide and conducting the analysis, demonstrating a systematic enquiry into acceptability, and contributing to the wider field as well as the topic area.
2. The length of the interviews facilitated an in-depth exploration of older adults and BASIL Support Workers' experiences.
3. Conducting the interviews by telephone whilst discussing feasibility of telephone delivery may have enabled contextual cues to be discussed that may have been missed in a face-to-face interview set up, however may have led to a self-selecting sample of people who were comfortable with the telephone.
4. A limitation is that the short timescale for the study meant that participants had to be interviewed as they completed 3m outcome measures, rather than using strategic sampling.

For peer review only

Acceptability of a behavioural intervention to mitigate the psychological impacts of COVID-19 restrictions in older people with long-term conditions: a qualitative study

Introduction

In England the number of people aged ≥65 years is projected to grow by 20% over the next decade, and by nearly 60% in 25 years.[1] Ageing is a risk factor for social isolation and loneliness. Social isolation is a quantifiable sense of reduced social network size and impoverished social contact.[2] Loneliness is the subjective psychological expression of social isolation owing to dissatisfaction with the frequency and quality of social contacts[3]. Social isolation and loneliness are established risk factors among older adults for cognitive impairment, depression and mortality.[4-6] In addition to the toll on the individual, loneliness impacts on health and social care services.[7] Loneliness is associated with an increased risk of subsequent depression, and this risk can persist for up to 12 years after the loneliness is reported.[8] One in four older people in the UK live with a mental health condition, most commonly depression, and the prevalence is higher among older people with multi-morbidities.[9] The COVID-19 pandemic and the associated loneliness and lack of social contact has impacted the mental health of older adults,[10] and the government's strategy on loneliness sets reducing social isolation and improving well-being among older adults as a public health priority.[11]

The Behavioural Activation in Social IsoLation (BASIL) study investigated the acceptability and feasibility of a remotely delivered brief psychological intervention (Behavioural Activation, BA) to prevent or reduce depression and loneliness in older people with long term conditions during isolation (or shielding).[12] The study was adopted by the NIHR Urgent Public Health programme in May 2020.[13] BA works on the principle that low mood may be a consequence of inactivity, and loss of positive reinforcement due to a reduction in valued, pleasurable activities.[14] A bespoke intervention was used in the BASIL study incorporating BA, a self-help booklet and trained BASIL Support Workers, with the aim of encouraging the older adult to recognise the link between mood and behaviour, and reinstate or replace valued activities that generate positive reinforcement and so improve mood.

For peer review only

The BASIL pilot study findings have been reported elsewhere.[12] This paper reports the findings from the qualitative study within BASIL, presenting analysis using the Theoretical Framework of Acceptability (TFA).[15]

Methods

We conducted a nested qualitative study, using semi-structured interviews to explore the acceptability and feasibility of the BA intervention from the perspectives of older adult participants and BASIL Support Workers.

Ethical approval was provided by Yorkshire and The Humber - Leeds West Research Ethics Committee ref: 18/YH/0380 (approved as substantial amendment 02 under existing NIHR IRAS249030 research programme). The protocol for the BASIL pilot study was preregistered (ISRCTN94091479) on June 9, 2020.

Participant Selection

Sixteen study participants were interviewed from a pool of 86 study participants from the BASIL pilot study who consented to take part in an interview at initial recruitment (inclusion criteria: aged 65 years or over with two or more physical LTCs). All nine BASIL Support Workers consented to participate in an interview. Study participants were approached via telephone following completion of the intervention and one-month post-randomisation follow-up measures. Participants were contacted by the researcher (CS) and interviews were arranged at a time to suit the participant. BASIL Support Workers provided their verbal permission to be contacted followed completion of the BASIL intervention training. BASIL Support Workers were provided with study information and invited to contact the interviewing researcher if they were willing to participate in an interview.

Data Collection

Semi-structured interviews were conducted by telephone. The interviews took place between September and November 2020. Topic guides explored intervention delivery context (e.g., impact of the COVID-19 context on older adults), study processes (e.g. remote recruitment, mode of intervention delivery) and thoughts around intervention content (e.g., study materials). Study topic guides were sensitised by the seven constructs of the Theoretical Framework of Acceptability (TFA)[15] (Table 1).

For peer review only

Interviews were digitally-recorded using an encrypted dictaphone and transcribed verbatim by a professional transcribing company. The 'completer' interviews lasted between 30 and 56 minutes, and BASIL Support Worker interviews 39 and 60 minutes.

Analysis

We initially carried out an iterative thematic analysis,[16] so that we were able to fully explore and familiarise ourselves with the data (reported elsewhere[12]). We then used the theoretical derived Theoretical Framework of Acceptability (TFA)[15], which has 7 domains, described in Table 1.

Table 1 Overview of TFA Domains

Component	Definition
Affective Attitude	How an individual feels about the intervention
Burden	The perceived amount of effort that is required to participate in the intervention
Ethicality	The extent to which the intervention has good fit with the individual's value system
Intervention Coherence	The extent to which the participant understands the intervention and how it works
Opportunity Costs	The extent to which benefits, profits or values must be given up to engage in the intervention
Perceived Effectiveness	The extent to which the intervention is perceived as likely to achieve its purpose
Self-efficacy	The participant's confidence that they can perform the behaviour(s) required to participate in the intervention

Table 1. The seven-component construct of the Theoretical Framework of Acceptability, adapted from Sekhon, Cartwright & Francis (2017).

Using a theoretically derived framework specifically designed for assessing the acceptability of health care interventions was considered appropriate for this study, alongside a thematic analysis to provide a multi-dimensional assessment of the acceptability of the BA intervention and pilot study processes. Though TFA is a fairly novel method, examining acceptability through seven dimensions comprising acceptability (Table 1) ensures that factors are identified to inform the retrospective and prospective acceptability of BA as a healthcare intervention in older adults with LTCs, and thus inform a future trial.

Familiarisation of data relevant to the TFA was undertaken by drawing on the meaning of the domains[15] and then indexing data considered relevant to these domains using NVIVO 12.

For peer review only

This analysis was carried out using Constant Comparison[17] methods and regular research meetings were conducted to agree interpretation, relevance and meaning of indexing to the TFA domains. Data were then analysed by CS and AH within the TFA domains, initially by participant group. Constant Comparison methods were then applied to compare and contrast data across participant groups (data mapping) by CS. The Constant Comparison[17] method facilitated the development of categories of phenomena within the TFA domains that allowed for interpretation of participants' perceptions of the acceptability of the BASIL study. Final data interpretation was agreed through discussion with CS, AH, CCG and PC.

Patient and Public Involvement and Engagement

Stakeholder groups (with older adults, carers, clinicians and third sector practitioners) had been conducted pre-pandemic to develop the BA intervention and then online in April 2020 to refine the intervention for remote delivery. The public-facing documents were discussed with our patient participation group prior to submission for ethics approval. The findings and analysis from the qualitative study were discussed with the patient participation group.

Results

Participants

Semi-structured interviews were conducted with 15 participants who had completed the BA intervention ('completers'), and one with a participant who did not complete the intervention ('non-completer'). Completer participants had between 2-8 of a maximum 8 intervention sessions (mean = 6.5). Participant demographics for the pilot trial are reported elsewhere[12] along with a summary of the thematic analysis (see also Appendix 1).

Nine interviews were carried out with the BASIL Support Workers who delivered the intervention (characteristics reported elsewhere[12]).

Themes from the Thematic analysis are presented in Appendix 1.

Findings from the TFA analysis

This paper focuses on our findings within the Theoretical Framework of Acceptability (TFA), incorporating both older adult and BASIL Support Worker perspectives. Throughout the paper and Appendix 1, illustrative data is given with participant identifiers. Older adult

For peer review only

intervention 'completer' quotes are labelled with the prefix 'OA', then their identifier (e.g. OA09). The intervention 'non-completer' participant is labelled with the prefix 'OA', then 'NC' (NC: non-completer), before their identifier (e.g. OA NC07). BASIL Support Worker quotes are labelled with the prefix 'BSW', then their identifier (e.g., BSW03). The findings from the TFA analysis are presented under each TFA domain.

Affective Attitude

Affective attitude describes 'How an individual feels about the intervention'[15]. Affective attitude is described here in terms of the overall retrospective attitude to receiving or delivering the BASIL intervention. Older adults described a positive affective attitude towards the BASIL intervention:

> *I feel good about taking part. It's definitely been good for me, and I hope, if this gets off the ground, that it's going to be good for other people. It was a benefit for me, it really was a big benefit to me. (OA01)*

BASIL Support Workers were positive about being part of the BASIL intervention:

> *And I want to make a difference to people, sort of, experience of healthcare and make it more positive and the best, you know, they can have. So, it felt good to be involved in something that was trying to do that (BSW03)*

Some BASIL Support Workers described positive affective attitude in terms of adapting the study in response to the COVID-19 pandemic:

"*The study was adapted to BASIL to respond to COVID it felt so worthwhile and I felt really motivated to work really, really hard on it" (BSW05).*

It appeared, however, that a positive affective attitude could be attenuated with study challenges, such as the difficulties with activity planning in the context of COVID-19 restrictions:

> *The constant changes in rules and the confusion that people have had over that, has, you know…it has affected it [intervention delivery]. Because one week,*

For peer review only

someone can be doing okay and the next week, rules have changed and they're feeling anxious, so they're not really sure where they stand anymore. Or, you know, restrictions have been lifted… and they've been able to go back to doing most of what they normally do, and actually, they're loads better. So you really don't know from one week to the next, where someone's going to be at. (BSW07)

Some older adult participants discussed how they had valued the BASIL Support Worker being able to help with problem-solving and information-finding:

I suppose only that if I did have a problem, there was someone there that I could discuss it with, and someone who could perhaps help me, who knew the right people to contact (OA13).

Intervention Coherence

The coherence domain of the TFA is described as *'the extent to which the participant understands the intervention, and how the intervention works'*[15]. The overall aims of the BASIL Pilot study appeared coherent to both BASIL Support Workers and older adults:

The aims are to try and help them to find ways of coping with life events such as a pandemic. But not just that. If you can teach them this behavioural activation cycle thing then they can apply it to any life event or situation that's affecting them and give them a coping strategy. (BSW08)

I presume, or this is how I looked at it, was that you were trying to get information on how people were reacting within the COVID environment, and that you were hoping to get strategies out of it, that would then help people to cope better with the situation. (OA12)

BASIL Support Workers described the ease with which they could make links between activity and low mood, which underpins the BA part of the BASIL intervention. BSW09 articulates how they think the BA Intervention works, which also informs the 'perceived effectiveness' TFA domain:

For peer review only

> *So I think it's [BASIL intervention] set up as a structured guided support really, so that they'll get regular contact with one of the support workers who will look to help them to engage with the booklet, and maybe look at how they can make changes to what they're currently doing, in the hope that that's going to impact how they feel physically in terms of their health, but also emotionally (BSW09)*

Older adults appeared to have a good understanding of the relationship between activity, mood and physical health, suggesting that the BA intervention was understandable to participants and had a reasonable level of face validity:

> *I think they link greatly because obviously if you're doing something that keeps your…makes you feel good and keeps your mind away from anxieties then that's obviously going to help you feel better, it's going to help you face each day in a better way. (OA09)*

However, it appeared that the coherence of the intervention could be reduced when working with the BA cycle with participants who were not experiencing low mood:

> *Some* [older adults] *just said because they didn't have a low mood it didn't affect their behaviour. It was very difficult to try and make them see the cycle when they didn't see any relevance for them because they were fine. (BSW08)*

BSWs reflected that when people did not perceive themselves as having low mood, the BA intervention made less sense to them and they did not see how BA could work for them.

<u>Opportunity Cost</u>

Opportunity cost describes the '*extent to which benefits, profits, or values must be given up to engage in an intervention*'[15]. Most BASIL Support Workers talked about how their usual working role had been adjusted to accommodate the delivery of the BA intervention. As such intervention delivery had little cost to their usual working role:

For peer review only

P: Yeah, my caseload was adjusted slightly, so I had a couple of client contacts less my caseload for the week to create the time to be able to work on the study.

I: Did that work okay?

P: Yeah, it was absolutely fine, I was really supported by my [work] to be able to do that, they've been brilliant. (BSW04)

Other BASIL Support Workers described how intervention delivery fitted in with their working routine: *I just fitted it in with my diary and that worked fine (BSW07).*

Generally older adults described a low opportunity cost in terms of taking part in the BA intervention. This was attributed, in part, to having time available: *It wasn't bothering my timetable or anything like that, cause with the lockdown I've been doing very little anyway (OA11).* Other older adults described how the intervention could be planned flexibly around their other activities:

I did, just work it in and around my life. If there was something that had to be wrote down, then I would sort of spend perhaps an hour thinking about it, and then I'd go on and do something else, and then I'd come back to it, and spend another hour maybe writing down what I wanted to say and that, you know (OA12)

<u>Burden</u>

The TFA domain of Burden describes 'The perceived amount of effort that is required to participate in the intervention'[15]. Some BASIL Support Workers described a time burden associated with delivering the intervention sessions:

If somebody lives on their own and the social isolation is more of a problem, then it's really hard to keep it to 30 minutes and generally they take an hour, those calls. (BSW06).

One BASIL Support Worker discussed the emotional effort involved in delivering the intervention in the pandemic, due to the social restrictions that everyone faced, and reported that it could be an effort to stay positive about the future:

For peer review only

It was just everything was just a bit more of an effort but that was the world per se really. It was for everybody and anybody that was working everything was more difficult, yeah. (BSW01)

Some older adults felt the amount of effort required to take part in the BASIL study was minimal, whilst others described the mental effort involved in taking part in the intervention. The data extracts below illustrate these contrasting views:

There was a little bit of effort. I mean obviously from week to week you had these tasks, your diary to fill in and to think about the tasks that you were doing that weren't enjoyable and how I could break it down into smaller chunks rather than do the whole thing in one go…So there were some things to do but it wasn't onerous at all. (OA03)

No physical effort whatsoever. Mental effort is the problem. And just, I didn't want to let the [person] who was doing the questioning, down, so I used to have to think about what was required, you know, and what [they] was expecting from me, and then try and adapt it into it. So, yes, there was a bit of effort involved, from the point of view that you don't want to do it, but you have to do it, to keep things on an even track, you know? (OA12)

<u>Self-efficacy</u>

The Self-efficacy domain of the TFA describes '*The participant's confidence that they can perform the behaviour(s) required to participate in the intervention*'[15]. Here, Self-efficacy is described in terms of how confident both BASIL Support Workers and older adults felt undertaking the BASIL intervention overall.

Some BASIL Support Workers described how, over time, their confidence to deliver the intervention grew with experience:

I think your confidence definitely grows with familiarity, so the first few times you're having a session with a participant, you're very keen to make sure you've got everything, all your materials to hand, that you know what you're

For peer review only

doing and what's expected of you, making sure that you're following the protocol. As it all starts to become more familiar and you can really enjoy using the booklet, it's really nice to have such nice materials to use with the client and the participant. (BSW04)

Some participants expressed a degree of increased confidence, satisfaction and mastery in having completed the intervention:

I wouldn't say confident, but I did learn a lot, and I think at the end of it, I did feel more confident. At the end of it all, I thought, well, I've done that and I'm much better off for doing it. (OA01)

Others were, however, more equivocal about engaging with the intervention but still recognised that they had become more certain about coping with the pressures of social isolation:

I didn't feel unconfident. I felt apprehensive, maybe, you know, it's all this…is this really Big Brother keeping their eye on us, blah blah blah. But I think everybody I've spoken to has been really friendly and helpful and it's helped draw me out and helped me see things better and cope with things better. No, I don't think I felt unconfident, just more apprehensive (OA14)

<u>Perceived effectiveness:</u>

Perceived effectiveness describes *'The extent to which the intervention is perceived as likely to achieve its purpose'*[15.] During the interviews, we asked if participants thought the BASIL study would achieve its aims, and to gauge how positive they felt about this, we also asked if participants would '*recommend the study to others*'[18].

Older adults reflected that the BASIL study would achieve its aims:

It does cover quite a lot really. As I say, it seems to be, you know, centred obviously towards mood and how the…this pandemic has affected how people feel and there, sort of, fears and what have you. So yes, I think it's very good (OA09)

For peer review only

BASIL Support Workers also acknowledged that BA had the potential to support people who were shielding and isolated during the pandemic:

> *I hadn't thought about it [BA], in terms of delivering for COVID. And I think, yeah, it's a really good approach for this. Obviously, I've never thought about it before, I've not been in a global pandemic before, but yeah, I think it's a useful intervention. (BSW07)*

Both older adults and BASIL Support Workers reflected that the effectiveness of the BASIL intervention was likely to be enhanced if it was more specifically targeted at those with low mood and long-term physical health problems:

> *I think possibly it needs to be more targeted, so anybody who has painful medical condition or who lives alone who is isolated, certainly I think it would benefit them a lot. I think the wide spread that you've currently can be more targeted and more focused and more helpful to more people in that sense. (OA02)*

> *I think, yes, but I do think just some consideration needs to be given to who we're targeting, maybe it's not quite so useful for people on the threshold of depression and feel that they're doing quite well………..... I wonder if perhaps it would be more beneficial just to focus on people who are expressing their own thoughts of feeling low. (BSW04)*

Most participants, (Support Workers and older adults), said they would recommend the BASIL study to others:

> *Yes, definitely. But either I'd like to think that perhaps an older family member who was struggling and there was an opportunity, who was struggling who knew there was an opportunity to participate in the BASIL study, I would say yes. I'd also say yes to my colleagues because I think it really helps. (BSW04)*

> *I think, well, I'd definitely recommend it to people who want to gain some experience with working with older adults, because it's great insight. And then for*

For peer review only

> *the older adults themselves, yeah, particularly those who are struggling with low mood, I can see that it is a really valuable resource and it's helpful to have that kind of contact, even if it just gets them talking about how they're feeling, because that might not be something that they've done previously. (BSW09)*

Ethicality

The Ethicality sub-theme describes '*The extent to which the intervention has a good fit with an individual's value system*'[15]. Ethicality was explored by enquiring what participants valued most or least about the BASIL intervention. BASIL Support Workers often described how they had valued being able to help older people during the pandemic:

> *I think most valid was just getting to know and help older adults, particularly at this time where we know mental health, particularly things like depression and things like that, has risen. So that's been [inaudible 53:15], I just like the fact that I was able to help this group of people even if it is only [number] of them I've managed to help. (BSW 06)*

Other older adults described feeling positive about the BASIL intervention, primarily because it presented an opportunity to be altruistic:

> *I:How do you feel about taking part in the BASIL Support Package?*
> *P:I think if it's helping the likes of yourself to understand how people are managing that if I've been able to be a part of that and assist in any way I'm pleased to have done it.(OA07)*

One older adult appeared to value the social contact the intervention provided:

> *I think, it's actually nice to speak to people that are doing this because I think it's…in the present situation, I think anybody who's doing something like you are doing, I think is pretty good, I think it's wonderful and it's great that you're doing it, you know, for whatever reason (OA09)*

Others discussed personal gains made from taking part in the intervention overall:

For peer review only

The thing I valued most is that it has made me more reflective and more grateful and that's the thing that I got out of it that it made me really look at my life and re-think how I do things (OA02)

Discussion

The BASIL Pilot study was designed to test the feasibility and acceptability of recruiting and remotely delivering a BA intervention to older adults with two or more LTCs during the COVID-19 pandemic. The intervention was set within a collaborative care framework and was designed to prevent and reduce depression and loneliness in socially isolated older adults. The initial thematic analysis (TA) (Appendix 1) suggested that the recruitment procedures to the BASIL pilot trial and the BA intervention (including the self-help booklet) were acceptable. Findings from the TA[12] informed the protocol for the definitive BASIL+ randomised controlled trial.[22]. Remote delivery of the intervention was acceptable and the BA self-help booklet was engaging and relevant, but less so for those without low mood. Activity planning during the intervention was difficult whilst COVID-19 restrictions were in place. Whilst altruism facilitated study participation, the BASIL trial needs to be targeted to those with depressive symptoms. TA highlighted detail surrounding the intervention context; variability in how the restrictions had impacted participants' mental health and perceived lack of access to primary care which could influence the way participants felt about the intervention but were not picked up by the TFA.

The TFA[15] added to the TA analysis and provided a comprehensive framework to inform the retrospective and prospective acceptability of the BA intervention. All seven of the TFA component constructs were populated with data from the interview transcripts and provided insights that were not identified by the initial TA. However, the ethicality component was more difficult to code, and sometimes overlapped with the Affective Attitude component. Confidence in the intervention (Self-Efficacy) grew over time for both older adults and BASIL Support Workers. For older adults, previous experience of similar interventions promoted self-efficacy, and some older adults reported greater self-efficacy as they experience positive effects from taking part. For BASIL Support Workers, self-efficacy grew with experience of delivering the intervention, although was reduced by having a long gap between training and intervention delivery. Both older adults and Support Workers valued the opportunity to demonstrate altruism in the context of the pandemic, but older adults also

valued the social contact and assistance with problem solving and seeking healthcare provision. The intervention had low opportunity cost for both Support Workers and older adults, who found delivering and taking part in the intervention fitted in around other commitments.

Theoretical Constraints of TFA

Burden and opportunity cost were the least populated domains – it could be that low perceived burden and low opportunity cost means participants elaborate less on these topics, or it could be that the volume and richness of the data coded to other domains indicates that those elements appealed more to participants compared to the burden or opportunity cost of the BASIL intervention. Ethicality was a difficult component to evaluate and was closely related to Affective Attitude. The questions "Is there anything you valued about the BASIL study intervention?" and "Would you recommend the BASIL study to others?" were used to target Ethicality, but it was sometimes difficult to separate participants talking about what they valued versus talking about their value system; often participants described liking the intervention for what it can offer and the benefits they gained, and it was left to the research team to interpret whether this was in line with their value system or an expression of how they felt. Perhaps Ethicality is easier to analyse in interviews with healthcare professionals with a clear set of professional values, compared to interviews with participants where their individual value system and core personal values are more implicit. Future iterations of the TFA should consider elaborating on the construct meanings and perhaps provide example questions that researchers can adopt in topic guides. The TFA approach has added value, justification for concluding that the intervention is acceptable, and provided insights into how to refine the recruitment processes for a definitive main BASIL trial (BASIL+ ISRCTN63034289) and is comprehensive enough to evaluate acceptability alone if time and resources are limited, however, carrying out an inductive TA first elucidated important contextual information that would have otherwise been missed, therefore we would recommend doing both.

Telephone delivery of the intervention was acceptable, although some older adults suggested that if it were not for the pandemic, they would have preferred face-to-face delivery. This finding fits with previous work in the acceptability of telephone-based therapy for depression. Despite user ambivalence, there is no clear evidence that the use of the telephone negatively affects the interactional elements of therapy.[19] Furthermore, telephone-delivered case

For peer review only

management has proven efficacy in supporting the implementation of multidisciplinary approaches to managing depression, such as collaborative care.[20] our findings offer further support for the utility of using the telephone to deliver low-intensity psychological interventions, even where there might be an expressed preference for face-to-face approaches.

To conclude, this nested qualitative study explored the perspectives and experiences of those involved in delivering and participating in the BASIL pilot study.[12] Analysis using the TFA[15] provides a novel analytic lens to understand key aspects of acceptability, but an initial TA is valuable. To our knowledge this is the first process evaluation of a trial and intervention designed to mitigate the psychological impact of COVID-19, addressing the research priority of evaluating brief psychosocial interventions to prevent depression and loneliness in vulnerable populations during the COVID-19 pandemic.[21] This contributes to the evidence around psychosocial and behavioural interventions for older adults addressing the mental health impact of COVID-19 and beyond.

For peer review only

Ethics Approval

This study involves human participants and ethical approval was provided by Yorkshire and The Humber - Leeds West Research Ethics Committee ref: 18/YH/0380 (approved as substantial amendment 02 under existing NIHR IRAS249030 research programme). Participants gave informed consent to participate in the study before taking part.

Contributorship Statement

Author contributions can be found in Appendix 2.

Competing Interests

We have read the journal's policy and the authors of this manuscript have the following competing interests: DE and CCG are current committee members for the NICE Depression Guideline (update) Development Group, and SGi was a member between 2015-18. SGi, PC and DMcM are supported by the NIHR Yorkshire and Humberside Applied Research Collaboration (ARC) and DE is supported by the North East and North Cumbria ARCs.

Funding

This work is funded by funded by National Institute for Health Research (NIHR) Programme Grants for Applied Research (PGfAR) RP-PG-0217-20006 The funder had no role in study design, data collection and analysis, decision to publish, or preparation of the manuscript.

Data sharing statement

Data are available on reasonable request. All data relevant to the study are included in the article or uploaded as online supplemental information. The datasets used and analysed during the current study are available from the corresponding author on reasonable request.

For peer review only

References:

1. Office for National Statistics. Living longer: how our population is changing and why it matters. Overview of population ageing in the UK and some of the implications for the economy, public services, society and the individual, 2018.
2. de Jong Gierveld J, Havens B. Cross-national Comparisons of Social Isolation and Loneliness: Introduction and Overview. *Canadian Journal on Aging / La Revue canadienne du vieillissement* 2004;23(2):109-13. doi: 10.1353/cja.2004.0021 [published Online First: 2010/03/31]
3. Peplau LA. Loneliness Research: Basic Concepts and Findings. In: Sarason IG, Sarason BR, eds. Social Support: Theory, Research and Applications. Dordrecht: Springer Netherlands 1985:269-86.
4. Cacioppo JT, Hughes ME, Waite LJ, et al. Loneliness as a specific risk factor for depressive symptoms: Cross-sectional and longitudinal analyses. *Psychology and Aging* 2006;21(1):140-51. doi: 10.1037/0882-7974.21.1.140
5. Eng PM, Rimm EB, Fitzmaurice G, et al. Social ties and change in social ties in relation to subsequent total and cause-specific mortality and coronary heart disease incidence in men. *Am J Epidemiol* 2002;155(8):700-9. doi: 10.1093/aje/155.8.700 [published Online First: 2002/04/12]
6. Shankar A, Hamer M, McMunn A, et al. Social isolation and loneliness: relationships with cognitive function during 4 years of follow-up in the English Longitudinal Study of Ageing. *Psychosom Med* 2013;75(2):161-70. doi: 10.1097/PSY.0b013e31827f09cd [published Online First: 2013/01/31]
7. Dreyer K, Steventon A, Fisher R, et al. The association between living alone and health care utilisation in older adults: a retrospective cohort study of electronic health records from a London general practice. *BMC Geriatrics* 2018;18(1):269. doi: 10.1186/s12877-018-0939-4
8. Lee, S. L., Pearce, E., Ajnakina, O., Johnson, S., Lewis, G., Mann, F., ... & Lewis, G. (2021). The association between loneliness and depressive symptoms among adults aged 50 years and older: a 12-year population-based cohort study. *The Lancet Psychiatry*, *8*(1), 48-57.
9. Age UK (2019) Later Life in the United Kingdom 2019. *Age UK.* https://www.ageuk.org.uk/globalassets/age-uk/documents/reports-and-publications/later_life_uk_factsheet.pdf
10. Independent Age (2021) The Mental Health Experiences of Older People During the Pandemic. *Mental Health Foundation and Independent Age.* Accessed via: https://www.independentage.org/mental-health-experiences-of-older-people-during-pandemic
11. HM Government. A connected society. A strategy for tackling loneliness – laying the foundations for change, 2018.
12. Gilbody S, Littlewood E, McMillan D, Chew-Graham CA, Bailey D, et al. (2021) Behavioural activation to prevent depression and loneliness among socially isolated older people with long-term conditions: The BASIL COVID-19 pilot randomised controlled trial. PLOS Medicine 18(10)
13. NIHR (2022) Study detail: BASIL-C19. Accessed via: https://www.nihr.ac.uk/covid-studies/study-detail.htm?entryId=249030
14. Ekers D, Webster L, Van Straten A, Cuijpers P, Richards D, et al. (2014) Behavioural Activation for Depression; An Update of Meta-Analysis of Effectiveness and Sub Group Analysis. PLOS ONE 9(6).
15. Sekhon, M., Cartwright, M. & Francis, J.J. (2017) Acceptability of healthcare interventions: an overview of reviews and development of a theoretical

For peer review only

framework. *BMC Health Service Research* 17, 88

16. Braun, V., & Clarke, V. (2006). Using thematic analysis in psychology. *Qualitative Research in Psychology, 3*(2), 77–101. https://doi.org/10.1191/1478088706qp063oa
17. Glaser, B. G. (1965). The constant comparative method of qualitative analysis. Social problems, 12(4), 436-445.
18. Marshall et al (2015). The HELPER programme: HEalthy Living and Prevention of Early Relapse – three exploratory randomised controlled trials of phase-specific interventions in first-episode psychosis. Appendix 5. The InterACT trial acceptability interview topic guide. https://www.ncbi.nlm.nih.gov/books/NBK285595/
19. Irvine A, Drew P, Bower P, et al. Are there interactional differences between telephone and face-to-face psychological therapy? A systematic review of comparative studies. *Journal of Affective Disorders* 2020;265:120-31. doi: https://doi.org/10.1016/j.jad.2020.01.057
20. Hudson JL, Bower P, Kontopantelis E, et al. Impact of telephone delivered case-management on the effectiveness of collaborative care for depression and anti-depressant use: A systematic review and meta-regression. *PLOS ONE* 2019;14(6):e0217948. doi: 10.1371/journal.pone.0217948
21. Holmes, EA., O'Connor, RC., Perry, VH. et al. (2020) Multidisciplinary research priorities for the COVID-19 pandemic: a call for action for mental health science. Lancet Psychiatry. 7: p547-560.
22. Burke L, Littlewood E, Gascoyne S, McMillan D, Chew-Graham CA, Bailey D, et al. (2022) Behavioural Activation for Social IsoLation (BASIL+) trial (Behavioural activation to mitigate depression and loneliness among older people with long-term conditions): Protocol for a fully-powered pragmatic randomised controlled trial. PLoS ONE 17(3).

For peer review only

License Statement

I, the Submitting Author has the right to grant and does grant on behalf of all authors of the Work (as defined in the below author licence), an exclusive licence and/or a non-exclusive licence for contributions from authors who are: i) UK Crown employees; ii) where BMJ has agreed a CC-BY licence shall apply, and/or iii) in accordance with the terms applicable for US Federal Government officers or employees acting as part of their official duties; on a worldwide, perpetual, irrevocable, royalty-free basis to BMJ Publishing Group Ltd ("BMJ") its licensees and where the relevant Journal is co-owned by BMJ to the co-owners of the Journal, to publish the Work in BMJ Open and any other BMJ products and to exploit all rights, as set out in our licence.

The Submitting Author accepts and understands that any supply made under these terms is made by BMJ to the Submitting Author unless you are acting as an employee on behalf of your employer or a postgraduate student of an affiliated institution which is paying any applicable article publishing charge ("APC") for Open Access articles. Where the Submitting Author wishes to make the Work available on an Open Access basis (and intends to pay the relevant APC), the terms of reuse of such Open Access shall be governed by a Creative Commons licence – details of these licences and which Creative Commons licence will apply to this Work are set out in our licence referred to above.

For peer review only

Appendix 1 Themes from thematic analysis (TA)

Theme	Descriptive summary of theme	Illustrative data extracts
Intervention context	The COVID-19 restrictions applied variably to older adults in the BASIL study (e.g., shielding, social distancing) dependent on their health status. There was considerable variability in how the restrictions had impacted participants' mental health.	*I feel as though I'm held in, I'm being persecuted because I was shielding originally. I did everything that was asked of me, we're now moving out but all of a sudden because of political decisions, I'm now finding myself back where I was. Staying in a lot, don't meet a lot of people at the best of times, generally speaking you'll probably say loneliness, isolation. (OA06)*<br><br>*But I just didn't feel motivated to do anything 'cause all the things that I really had been enjoying had just suddenly stopped overnight, literally*. (OA14)<br><br>*It's just a worry, obviously I don't want to catch it, you know, because of my underlying conditions I think I would have a rough time of it, you know …So, I'm quite happy to isolate myself, if you like (OA12)*<br><br>*The constant changes in rules and the confusion that people have had over that, has, you know…it has affected it [intervention delivery]. Because one week, someone can be doing okay and the next week, rules have changed and they're feeling anxious, so they're not really sure where they stand anymore. Or, you know, restrictions have been lifted. And so one week, they're really struggling and the next week they've been able to*

For peer review only

		go back to doing most of what they normally do, and actually, they're loads better. So it changes week to week. So you really don't know from one week to the next, where someone's going to be at, dependent on, you know, the restrictions in their area. (BSW07)
	Remote consulting / perceived lack of access to primary care	*My diabetes, I think, they've forgot about me, I have no idea I haven't…the last two appointments were supposed to be phone ones and they didn't mat…they were cancelled for different reasons. My cancer ones, I don't know what's happening there, I've missed the last two because of the same problem. They've rang up to say that either the person's not available or the computer's broke down, or…so that's my cancer and my diabetes. (OA11)*<br><br>*There's been these two sides of things. There's one side, it's not affected their long-term conditions or their access in the support. The other side is where they're not wanting to access the support because they're either too scared or they don't want to go out, and then that's having the impact on their long-term conditions 'cause they're not being able to go to their routine appointments for it and things like that. (BSW06)*
Study entry	Phased approach to recruitment (i.e.: 1) initial text from GP surgery, 2) letter with study participant information (sent via GP surgery), 3) phone call from the Clinical Research	*So, I was really quite down in the dumps a bit. And then I got this text from my doctor saying that I should take part in…[they] thought it would be beneficial for me to take part. So, I said yes, I would do that and I rang the number and said yes I would.* (OA01)

For peer review only

	Network and 4) phone call from the University study team), was both feasible and acceptable. It seemed to provide successively layered opportunities for participants to gain sufficient information and decide if they wanted to participate in the BASIL study.<br><br>Need for targeted recruitment.	*No, I mean the doctor gives you a warning that this is about to happen and then you're prepared when somebody phones up that it's not a scam, a con, which is what I don't worry about it because I know how to deal with it but to some people it could be worrying. (OANC17)*<br><br>*I think it's maybe thinking about who we would want to target in the main study. I think as I say, the participants I was working with didn't consider themselves to be struggling with their mood, they'd come into the study for altruistic reasons, rather than looking to boost their own mood and wellbeing. I guess maybe to be more targeted. (BSW04)*
Intervention delivery and content	The BASIL pilot intervention was solely delivered by telephone to older adults in their homes. Online intervention delivery was offered; however, this option was not taken up by any participants.<br><br>Duration of intervention	*I: receiving the support package over the telephone, how was that?*<br>*P: Well, I found that the person I was speaking with showed a lot of empathy, understood where I was coming from, you know, just about being on your own for long periods of time and that sort of thing. (OA06)*<br><br>*And I thought, I don't want to be bothered with not being able to get it [online platform] on, you know, I'd rather a telephone call and then I don't have the worry of the [online platform] going wrong. (OA15)*<br><br>*So if they know they've got a telephone call on a*

For peer review only

		Wednesday morning at 11, then that will really help them to structure their week and give them something to look forward to and mark their weeks. (BSW05)<br><br>*So for those who have more severe low mood, when I start working with them, I definitely see that they have more to learn in terms of using technology. (BSW06)*
	Self-help booklet	*I think it was enough because we'd done the whole book and it left me, at the end, to have an action plan. So that's all there, I'd written out on the page, so I've got that to refer to. Yes, I think eight weeks was enough. (OA15)*
	BA	*My first thought was, it was in very simple, precise language so that anybody could understand it. It wasn't, you know, sort of above anybody's head if you know what I mean. I thought it was very good, very simple. And it had different examples of what people were doing and how they were overcoming their difficulties. Yes, I thought it was very good, I liked it. (OA15)*<br><br>*And there was also plenty of opportunity for me to put my two pennorth worth in, you had pages that were blank and…list your three most priority items today……. You know, that was so…so it gave you that. (OA06)*

For peer review only

		with the booklet and the [person] who I was talking with, we were able to say what is it about you that's so important that you must have this priority and that priority, and they compete, why don't you break it down. What can I do, how much can I do, how does it interwork with each other, and it's that breaking down of… catastrophic how you are, obviously, the pandemic, how does the pandemic impact you, what can you do to circumvent it, you know, that sort of thing. And that was very useful. (OA06)<br><br>*Also, probably the breaking things down into making them more manageable. That's been particularly useful for physical health. So that helped with the physical pain. (BSW06)*<br><br>*And I felt really good to tell {them] that it made me feel better, it made me feel good. The support I got from [them] was really good because things that I'd never thought about, [they] said [themselves], why don't you…you know, have you tried this? (OA01)*

For peer review only

Appendix 2 Author Contributions using the CRediT Taxonomy

**Contributor Role**	**Role Definition**	
**Conceptualization**	Ideas; formulation or evolution of overarching research goals and aims.	CCG, PC, CS, EL, SGa
**Data Curation**	Management activities to annotate (produce metadata), scrub data and maintain research data (including software code, where it is necessary for interpreting the data itself) for initial use and later reuse.	CS, AH
**Formal Analysis**	Application of statistical, mathematical, computational, or other formal techniques to analyze or synthesize study data.	CS, CCG, PC
**Funding Acquisition**	Acquisition of the financial support for the project leading to this publication.	SG, DE, CCG, EL, PC, DM
**Investigation**	Conducting a research and investigation process, specifically performing the experiments, or data/evidence collection.	CS
**Methodology**	Development or design of methodology; creation of models	CCG, PC, CS, EL, SGa, LB, ER, AH, LS, DB, DM
**Project Administration**	Management and coordination responsibility for the research activity planning and execution.	CS, EL, CCG
**Resources**	Provision of study materials, reagents, materials, patients, laboratory samples, animals, instrumentation, computing resources, or other analysis tools.	N/A
**Software**	Programming, software development; designing computer programs; implementation of the computer code and supporting algorithms; testing of existing code components.	N/A
**Supervision**	Oversight and leadership responsibility for the research activity planning and execution, including mentorship external to the core team.	CCG, DE, SG, EL, CS
**Validation**	Verification, whether as a part of the activity or separate, of the overall replication/reproducibility of	EL, SG, DE

For peer review only

Contributor Role	Role Definition	
	results/experiments and other research outputs.	
**Visualization**	Preparation, creation and/or presentation of the published work, specifically visualization/data presentation.	LS, CCG, PC, EN, CS
**Writing – Original Draft Preparation**	Creation and/or presentation of the published work, specifically writing the initial draft (including substantive translation).	LS, CS
**Writing – Review & Editing**	Preparation, creation and/or presentation of the published work by those from the original research group, specifically critical review, commentary or revision – including pre- or post-publication stages.	LS, CCG, PC, AH, EN, SG, DE, DM, CS, ER, LB, SGa, DB, RW

For peer review only

Table 1
Consolidated criteria for reporting qualitative studies (COREQ): 32-item checklist

No	Item	Guide questions/description
**Domain 1: Research team and reflexivity**		
Personal Characteristics		
1.	Interviewer/facilitator	Which author/s conducted the interview or focus group?<br>**Claire Sloan**
2.	Credentials	What were the researcher's credentials? *E.g. PhD, MD*<br>**PHD in Psychology (University of Leeds) 2008, Graduate Diploma in Psychology (University of East London) 2009, Post Graduate Certificate in Low Intensity Psychological Interventions (University of Surrey) 2010, Post Graduate Diploma in Fundamentals of Neuroscience (Sheffield Hallam University) 2017**
3.	Occupation	What was their occupation at the time of the study?<br>**Research Fellow**
4.	Gender	Was the researcher male or female?<br>**Female**

No	Item	Guide questions/description
5.	Experience and training	What experience or training did the researcher have?<br>**Claire has extensive experience of the typical processes involved in health research, such as gaining REC and HRA approval, designing research protocols and recruiting/ interviewing vulnerable adults. She has also facilitated focus groups, PPI meetings, and analysed both qualitative and quantitative data for both her own research and studies she has assisted with. Claire has the additional experience of thesis supervision and facilitating qualitative research seminars, as well as Deaf Awareness Training.**
Relationship with participants		
6.	Relationship established	Was a relationship established prior to study commencement?<br>**No, this was avoided to eliminate possible bias**
7.	Participant knowledge of the interviewer	What did the participants know about the researcher? e*.g. personal goals, reasons for doing the research*<br>**Participants had little knowledge about the interviewer (CS), just that she was researcher on the study interested in hearing about their experience of the intervention.**
8.	Interviewer characteristics	What characteristics were reported about the interviewer/facilitator? e.g. *Bias, assumptions, reasons and interests in the research topic*<br>**N/A**

No	Item	Guide questions/description
**Domain 2: study design**		
Theoretical framework		
9.	Methodological orientation and Theory	What methodological orientation was stated to underpin the study? *e.g. grounded theory, discourse analysis, ethnography, phenomenology, content analysis*<br>**This research was based on two methodological approaches; the first was Thematic Analysis (TA) described by Braun and Clarke (2006) and the second was the Framework Method of Analysis, Gale et. al (2013), and its interpretation by Sekhon et.al (2017)**<br><br>**TA has 6 stages. Initial data familiarisation (stage 1) was carried out by Claire Sloan, Carolyn Chew-Graham and Peter Coventry, this involved reading initial interview transcripts and discussion of general observations within the context of the main research aims. More formal data coding (Stage 2) was carried out on completion of data collection, included all interview data and was carried out within participant groups using NVIVO 12. Coding was guided by the need to identify that information most relevant to the understanding of the operationalisation of the BASIL intervention. However, coding also offered the opportunity for 'data-driven coding (Braun & Clarke, 2006) in areas of discussion that were not a product of key topic guide prompted discussion. In stage 3 (generating themes), consideration of the meanings (Braun & Clarke, 2006) and relevance of coded data across participant groups resulted in the development of**

No	Item	Guide questions/description
		**an initial overarching thematic organisation of data across participant groups. Subsequent reviewing and refining of themes (Stages 4 and 5) was agreed by CS, PC and CCG. The final themes identified were as follows: 1: 'Intervention Context, 2: 'Study entry' and 3: Intervention delivery and content (stage 6).**<br><br>**In order to get feedback on the acceptability of the intervention, questions were guided by Sekhon et.al's (2017) interpretation of the Framework Method of Analysis, presented as the Theoretical Framework of Acceptability (TFA). The TFA has 7 domains. These are 1. Affective attitude (how the individual feels about the intervention), 2. Burden (the percieved amount of effort required to take part in the intervention), 3. Ethicality (the extent to which the intervention is a good fit with the individual's value system), 4. Intervention Coherence (the extent to which the individual understands the intervention) 5. Opportunity costs (the extent to which benefits, profits or values must be given up to take part in the intervention), 6. Perceived effectiveness (the extent to which the intervention is likely to achieve its purpose), and 7. Self efficacy (the participant's confidence that they can perform the behaviours required to engage in the intervention).**
Participant selection		
10.	Sampling	How were participants selected? *e.g. purposive, convenience, consecutive, snowball*<br>**N/A**

No	Item	Guide questions/description
11.	Method of approach	How were participants approached? e*.g. face-to-face, telephone, mail, email* **Intervention participants who consented to being invited to a qualitative interview were contacted by telephone in order to check they were still happy to take part, and to arrange the interview. Basil support workers who provided their consent to be interviewed at their training were emailed to arrange the interview.**
12.	Sample size	How many participants were in the study? **15 intervention participants who completed the intervention (completers), 1 participant who did not complete the intervention (non completers) and 9 BSW's were involved in this qualitative research.**
13.	Non-participation	How many people refused to participate or dropped out? Reasons? **N/A**
Setting		
14.	Setting of data collection	Where was the data collected? e*.g. home, clinic, workplace* **As with all data in the BASIL study, interviews were conducted remotely over the telephone.**
15.	Presence of non-participants	Was anyone else present besides the participants and researchers? **N/A (its possible that some interviewees had family members present but unlikely)**
16.	Description of sample	What are the important characteristics of the sample? *e.g. demographic data, date*

No	Item	Guide questions/description
		**The sample included both completers and non completers in an effort to understand their experience of the intervention, and why they might not have seen it through to the end. The addition of BSW provided more of a healthcare context as they described their clinical backgrounds and offered insight into various aspects of the intervention both positive and negative.**
Data collection		
17.	Interview guide	Were questions, prompts, guides provided by the authors? Was it pilot tested? **Questions were derived from the domains of the TFA and alongside these we included questions more specific to the intervention materials, recruitment process and the context of the intervention delivery (the COVID 19 pandemic and subsequent social restrictions). Prompts were included for those interviewees who might be having difficulty providing answers.**<br><br>**Although the questions were not pilot tested, we had recently conducted interviews with a similar population using the same methods for the MODS study so although the content of the questions differed, CS was well practiced in both the method of collection and interviewing older adults with long term health conditions.**
18.	Repeat interviews	Were repeat interviews carried out? If yes, how many?<br>**No**
19.	Audio/visual recording	Did the research use audio or visual recording to collect the data?

No	Item	Guide questions/description
		**Yes, an encrypted dictaphone was used to collect data from telephone interviews**
20.	Field notes	Were field notes made during and/or after the interview or focus group?<br>**N/A**
21.	Duration	What was the duration of the interviews or focus group?<br>**N/A**
22.	Data saturation	Was data saturation discussed?<br><br>**Yes - we planned to recruit no more than 15 intervention completers as we anticipated we would hit data saturation at this point. During analysis it did become apparent that this was the case especially with particular domains of the TFA such as Burden (widely reported to be low burden to intervention participants) and Opportunity costs (widely reported to not require any sacrifice on behalf of the participants).**
23.	Transcripts returned	Were transcripts returned to participants for comment and/or correction?<br>**No**
**Domain 3: analysis and findings**		
Data analysis		

No	Item	Guide questions/description
24.	Number of data coders	How many data coders coded the data?
25.	Description of the coding tree	Did authors provide a description of the coding tree?
26.	Derivation of themes	Were themes identified in advance or derived from the data?<br>**Derived from the data**
27.	Software	What software, if applicable, was used to manage the data?<br>**NVivo**
28.	Participant checking	Did participants provide feedback on the findings?<br>**No**
Reporting		
29.	Quotations presented	Were participant quotations presented to illustrate the themes / findings? Was each quotation identified? e*.g. participant number*<br>**Yes**
30.	Data and findings consistent	Was there consistency between the data presented and the findings?
31.	Clarity of major themes	Were major themes clearly presented in the findings?

No	Item	Guide questions/description
32.	Clarity of minor themes	Is there a description of diverse cases or discussion of minor themes?

For peer review only

BMJ Open

Acceptability of a behavioural intervention to mitigate the psychological impacts of COVID-19 restrictions in older people with long-term conditions: a qualitative study

Journal:	*BMJ Open*
Manuscript ID	bmjopen-2022-064694.R1
Article Type:	Original research
Date Submitted by the Author:	15-Dec-2022

Complete List of Authors:	Shearsmith, Leanne ; University of Leeds, Leeds Institute of Health Sciences<br>Coventry, Peter; University of York, Department of Health Sciences; University of York, York Environmental Sustainability Institute<br>Sloan, Claire; University of York, Department of Health Sciences<br>Henry, Andrew; University of York, Department of Health Sciences; Tees Esk and Wear Valleys NHS Foundation Trust, Research & Development<br>Newbronner, Liz; University of York<br>Littlewood, Elizabeth; University of York, Health Sciences<br>Bailey, Della; University of York, Health Sciences<br>Gascoyne, Samantha; University of York, Department of Health Sciences<br>Burke, Lauren; University of York, Department of Health Sciences<br>Ryde, Eloise; University of York, Department of Health Sciences; Tees Esk and Wear Valleys NHS Foundation Trust, Research & Development<br>Woodhouse, Rebecca; University of York<br>McMillan, Dean; University of York, Department of Health Sciences; Hull York Medical School<br>Ekers, David; University of York, Health Sciences; Tees Esk and Wear Valleys NHS Foundation Trust, Research & Development<br>Gilbody, Simon; University of York, Department of Health Sciences<br>Chew-Graham, Carolyn; Keele University, School of Medicine

**Primary Subject Heading**:	Mental health
Secondary Subject Heading:	General practice / Family practice, Geriatric medicine, Mental health, Public health
Keywords:	COVID-19, Depression & mood disorders < PSYCHIATRY, QUALITATIVE RESEARCH

SCHOLARONE™ Manuscripts

I, the Submitting Author has the right to grant and does grant on behalf of all authors of the Work (as defined in the below author licence), an exclusive licence and/or a non-exclusive licence for contributions from authors who are: i) UK Crown employees; ii) where BMJ has agreed a CC-BY licence shall apply, and/or iii) in accordance with the terms applicable for US Federal Government officers or employees acting as part of their official duties; on a worldwide, perpetual, irrevocable, royalty-free basis to BMJ Publishing Group Ltd ("BMJ") its licensees and where the relevant Journal is co-owned by BMJ to the co-owners of the Journal, to publish the Work in this journal and any other BMJ products and to exploit all rights, as set out in our licence.

The Submitting Author accepts and understands that any supply made under these terms is made by BMJ to the Submitting Author unless you are acting as an employee on behalf of your employer or a postgraduate student of an affiliated institution which is paying any applicable article publishing charge ("APC") for Open Access articles. Where the Submitting Author wishes to make the Work available on an Open Access basis (and intends to pay the relevant APC), the terms of reuse of such Open Access shall be governed by a Creative Commons licence – details of these licences and which Creative Commons licence will apply to this Work are set out in our licence referred to above.

Other than as permitted in any relevant BMJ Author's Self Archiving Policies, I confirm this Work has not been accepted for publication elsewhere, is not being considered for publication elsewhere and does not duplicate material already published. I confirm all authors consent to publication of this Work and authorise the granting of this licence.

For peer review only

Acceptability of a behavioural intervention to mitigate the psychological impacts of COVID-19 restrictions in older people with long-term conditions: a qualitative study

Leanne Shearsmith[1*], Peter A. Coventry[2], Claire Sloan[2], Andrew Henry[2,3], Elizabeth Newbronner[2], Elizabeth Littlewood[2], Della Bailey[2], Samantha Gascoyne[2], Lauren Burke[2], Eloise Ryde[2,3], Rebecca Woodhouse[2], Dean McMillan[2,4], David Ekers[2,3], Simon Gilbody[2,4], Carolyn A. Chew-Graham[5].

* Corresponding author: Leanne Shearsmith, Leeds Institute of Health Sciences, Room 10.38A Level 10, Worsley Building, University of Leeds, LS2 9NL. Email: l.shearsmith@leeds.ac.uk

1. Leeds Institute of Health Sciences, University of Leeds, Leeds, LS2 9NL, UK
2. Department of Health Sciences, University of York, York, YO10 5DD, UK
3. Tees, Esk and Wear Valleys NHS FT, Research & Development, Flatts Lane Centre, Middlesbrough, TS6 0SZ, UK
4. Hull York Medical School, University of York, York, YO10 5DD, UK
5. School of Medicine, Keele University, Staffordshire, ST5 5BG, UK

Keywords: COVID-19, Psychosocial Intervention, Qualitative Research, Behavior Therapy, Social Isolation
Word count: 5084

For peer review only - http://bmjopen.bmj.com/site/about/guidelines.xhtml

Abstract

Objectives

The COVID-19 pandemic heightened the need to address loneliness and social isolation (and associated incidence of depression) amongst older adults. Between June and October 2020, the Behavioural Activation in Social IsoLation (BASIL) pilot study investigated the acceptability and feasibility of a remotely delivered brief psychological intervention (Behavioural Activation) to prevent and reduce loneliness and depression in older people with long-term conditions during the COVID-19 pandemic.

Design

An embedded qualitative study was conducted with semi-structured interviews to generate data that was analysed inductively using thematic analysis and then deductively using the Theoretical Framework of Acceptability (TFA).

Setting

National Health Service and third sector organisations in England.

Participants

Sixteen older adults and 9 support workers participating in the BASIL pilot trial.

Results

Older adults and support workers described a positive affective attitude towards the intervention linked to altruism, however the activity planning aspect of the intervention was limited due to COVID-19 restrictions. The intervention was understood by older adults & support workers, although less understanding in older adults without low mood. A manageable burden was involved with delivering and participating in the intervention. For ethicality, older adults valued social contact and making changes, support workers valued being able to observe those changes. Opportunity cost was low for support workers & older adults. Behavioural Activation was perceived to be useful in the pandemic and likely to achieve its aims, (Perceived Effectiveness) especially if tailored to people with both low mood and long-term conditions. Self-efficacy developed over time and with experience for both support workers and older adults.

Conclusions

For peer review only - http://bmjopen.bmj.com/site/about/guidelines.xhtml

For peer review only

Overall, BASIL pilot study processes and the intervention were acceptable. Use of the TFA provided valuable insights into how the intervention was experienced and how the acceptability of study processes and the intervention could be enhanced ahead of the larger definitive trial (BASIL+).

Strengths & Limitations

1. The use of TFA in both informing the topic guide and conducting the analysis, demonstrating a systematic enquiry into acceptability, and contributing to the wider field as well as the topic area.
2. The length of the interviews facilitated an in-depth exploration of older adults and support workers' experiences.
3. Conducting the interviews by telephone whilst discussing feasibility of telephone delivery may have enabled contextual cues to be discussed that may have been missed in a face-to-face interview set up, however may have led to a self-selecting sample of people who were comfortable with the telephone.
4. A limitation is that the short timescale for the study meant that participants had to be interviewed as they completed 3-month outcome measures, rather than using strategic sampling.

For peer review only

Acceptability of a behavioural intervention to mitigate the psychological impacts of COVID-19 restrictions in older people with long-term conditions: a qualitative study

Introduction

Loneliness is the subjective psychological expression of social isolation owing to dissatisfaction with the frequency and quality of social contacts.[1] Social isolation is a quantifiable sense of reduced social network size and impoverished social contact.[2] Loneliness is an increasing problem across all age groups, including younger people.[3] However, loneliness is especially problematic in older adults because it is a risk factor for frailty,[4] which in turn, is a risk factor for depression.[5] The link between loneliness and subsequent depression can persist for up to 12 years after the loneliness is reported.[6] Social isolation and loneliness are also established risk factors among older adults for cognitive impairment and mortality.[7,8] In addition to the toll on the individual, loneliness impacts on health and social care services.[9]

In England the number of people aged ≥65 years is projected to grow by 20% over the next decade, and by nearly 60% in 25 years.[10] One in four older people in the UK live with a mental health condition, most commonly depression, and the prevalence is higher among older people with multi-morbidities.[11] The COVID-19 pandemic and the associated loneliness and lack of social contact has impacted the mental health of older adults.[12] Longitudinal data suggests that loneliness was an important predictor of increase in depressive symptoms in older adults during the pandemic, and that the negative mental health impacts of the pandemic persist and may worsen over the long term in the absence of effective interventions.[13] The government's strategy on loneliness sets reducing social isolation and improving well-being among older adults as a public health priority.[14]

The Behavioural Activation in Social IsoLation (BASIL) study investigated the acceptability and feasibility of a remotely delivered brief psychological intervention (Behavioural Activation) to prevent or reduce depression and loneliness in older people with long term conditions during isolation (or shielding).[15] The study was adopted by the NIHR Urgent Public Health programme in May 2020.[16] Behavioural Activation is an evidence-based psychological treatment. It works on the principle that low mood may be a consequence of

For peer review only
For peer review only - http://bmjopen.bmj.com/site/about/guidelines.xhtml

physical inactivity, and loss of positive reinforcement due to a reduction in valued, pleasurable activities.[17] It is a simpler psychological treatment than Cognitive Behavioural Therapy, but evidence suggests it can be an equally effective therapy for depression, even when delivered by junior mental health workers requiring less intensive and costly training.[18] Emerging evidence suggests that behavioural activation has an impact on depression and loneliness in the short term.[19] A bespoke intervention was used in the BASIL study incorporating Behavioural Activation, a self-help booklet and trained BASIL Support Workers, with the aim of encouraging the older adult to recognise the link between mood and behaviour, and reinstate or replace valued activities that generate positive reinforcement and so improve mood.

The BASIL pilot study findings have been reported elsewhere.[15,19] This paper reports the findings from the qualitative study within BASIL, presenting analysis using the Theoretical Framework of Acceptability (TFA).[20]

Methods

We conducted a nested qualitative study, using semi-structured interviews to explore the acceptability and feasibility of the intervention from the perspectives of older adult participants and BASIL Support Workers.

Ethical approval was provided by Yorkshire and The Humber - Leeds West Research Ethics Committee ref: 18/YH/0380 (approved as substantial amendment 02 under existing NIHR IRAS249030 research programme). The protocol for the BASIL pilot study was preregistered (ISRCTN94091479) on June 9, 2020.

Intervention

Behavioural activation was adapted with input from our patient and public advisory group. Support workers from a range of clinical and research backgrounds (Psychological wellbeing practitioners, mental health nurses, research assistants, clinical support officers, research nurses and crisis support workers) were trained to deliver the intervention by telephone or video call across 8 sessions. The intervention is described in more detail elsewhere.[19]

Participant Selection

Sixteen study participants were selected via consecutive sampling and interviewed from a pool of 86 study participants from the BASIL pilot study who consented to take part in an

For peer review only - http://bmjopen.bmj.com/site/about/guidelines.xhtml

interview at initial recruitment (inclusion criteria: aged 65 years or over with two or more physical long-term conditions). Two BASIL pilot study participants did not complete the intervention and one of these participants consented to interview as a non-completer. All nine BASIL Support Workers consented to participate in an interview. Study participants were approached via telephone following completion of the intervention and one-month post-randomisation follow-up measures. Participants were contacted by the researcher (CS) and interviews were arranged at a time to suit the participant. No relationship between participant and researcher was established prior to study commencement. Participants had minimal knowledge about the interviewer, and questions were not provided before the interview. Interviews were conducted by CS, a female research fellow with a PhD in Psychology. CS had extensive experience of interviewing and analysis of qualitative data, and Deaf Awareness training. BASIL Support Workers provided their verbal permission to be contacted followed completion of the BASIL intervention training. BASIL Support Workers were provided with study information and invited to contact the interviewing researcher if they were willing to participate in an interview.

For peer review only

Data Collection

One-to-one semi-structured interviews were conducted by telephone. The interviews took place between September and November 2020. Topic guides explored intervention delivery context (e.g., impact of the COVID-19 context on older adults), study processes (e.g. remote recruitment, mode of intervention delivery) and thoughts around intervention content (e.g., study materials). Study topic guides were sensitised by the seven constructs of the Theoretical Framework of Acceptability (TFA)[20] (Table 1).

Interviews were digitally-recorded using an encrypted dictaphone and transcribed verbatim by a professional transcribing company. The ‘completer' interviews lasted between 30 and 56 minutes, and BASIL Support Worker interviews 39 and 60 minutes. Field notes were not made during or after the interview. Transcripts were not returned to participants.

Analysis

We initially carried out an iterative thematic analysis,[21] so that we were able to fully explore and familiarise ourselves with the data (reported elsewhere[15]). We then used the theoretical derived Theoretical Framework of Acceptability (TFA)[20], which clearly articulates and defines a range of conceptually distinct constructs related to acceptability of interventions

For peer review only - http://bmjopen.bmj.com/site/about/guidelines.xhtml

(Table 1). These constructs go beyond operational definitions of acceptability such as drop-out and uptake rates of interventions which are often more aligned with notions of satisfaction than acceptability. Crucially the TFA can be used to capture responses about intervention acceptability from both recipients and those involved in delivering interventions. Additionally the TFA can be used to assess different aspects of acceptability before, during, or after intervention delivery, depending on intervention development and evaluation cycle.

Table 1 Overview of TFA Domains

Component	Definition
Affective Attitude	How an individual feels about the intervention
Burden	The perceived amount of effort that is required to participate in the intervention
Ethicality	The extent to which the intervention has good fit with the individual's value system
Intervention Coherence	The extent to which the participant understands the intervention and how it works
Opportunity Costs	The extent to which benefits, profits or values must be given up to engage in the intervention
Perceived Effectiveness	The extent to which the intervention is perceived as likely to achieve its purpose
Self-efficacy	The participant's confidence that they can perform the behaviour(s) required to participate in the intervention

Table 1. The seven-component construct of the Theoretical Framework of Acceptability, adapted from Sekhon, Cartwright & Francis (2017).

Using a theoretically derived framework specifically designed for assessing the acceptability of healthcare interventions was considered appropriate for this study, alongside a thematic analysis to provide a multi-dimensional assessment of the acceptability of the intervention and pilot study processes. We used the TFA after intervention delivery to elicit responses about the anticipated acceptability of the content and delivery of the BASIL intervention with a view to informing refinement of intervention features in advance of definitive evaluation.

Familiarisation of data relevant to the TFA was undertaken by drawing on the meaning of the domains[20] and then indexing data considered relevant to these domains using NVIVO 12. This analysis was carried out using Constant Comparison[22] methods and regular research meetings were conducted to agree interpretation, relevance and meaning of indexing to the TFA domains. Data were then analysed by CS and AH within the TFA domains, initially by participant group. Constant Comparison methods were then applied to compare and contrast

For peer review only - http://bmjopen.bmj.com/site/about/guidelines.xhtml

data across participant groups (data mapping) by CS. The Constant Comparison[22] method facilitated the development of categories of phenomena within the TFA domains that allowed for interpretation of participants' perceptions of the acceptability of the BASIL study. Final data interpretation was agreed through discussion with CS, AH, CCG and PC. Participants did not provide feedback on the findings.

Patient and Public Involvement and Engagement

Stakeholder groups (with older adults, carers, clinicians and third sector practitioners) had been conducted pre-pandemic to develop the intervention and then online in April 2020 to refine the intervention for remote delivery. The public-facing documents were discussed with our patient advisory group prior to submission for ethics approval. The questions were pilot tested with an individual from the patient advisory group. The findings and analysis from the qualitative study were discussed with the patient participation group.

Results

Participants

Semi-structured interviews were conducted with 15 participants who had completed the intervention ('completers'), and one with a participant who did not complete the intervention ('non-completer'). Data saturation was reached with 15 completer participants,[23] who had between 2-8 of a maximum 8 intervention sessions (mean = 6.5). Participant demographics for the pilot trial are reported elsewhere[15] along with a summary of the thematic analysis (see also Appendix 1).

Nine interviews were carried out with the BASIL Support Workers who delivered the intervention (characteristics reported elsewhere[15]).
No repeat interviews were carried out.
Themes derived from the data using Thematic analysis (Intervention context, Study entry, Intervention delivery and content) are presented in Appendix 1.

Findings from the TFA analysis

This paper focuses on our findings within the Theoretical Framework of Acceptability (TFA), incorporating both older adult and BASIL Support Worker perspectives. Throughout the paper and Appendix 1, illustrative data is given with participant identifiers. Older adult

For peer review only - http://bmjopen.bmj.com/site/about/guidelines.xhtml

intervention 'completer' quotes are labelled with the prefix 'OA', then their identifier (e.g. OA09). The intervention 'non-completer' participant is labelled with the prefix 'OA', then 'NC' (NC: non-completer), before their identifier (e.g. OA NC07). BASIL Support Worker quotes are labelled with the prefix 'BSW', then their identifier (e.g., BSW03). The findings from the TFA analysis are presented under each TFA domain.

Affective Attitude

Affective attitude describes 'How an individual feels about the intervention'[20]. Affective attitude is described here in terms of the overall retrospective attitude to receiving or delivering the BASIL intervention. Older adults described a positive affective attitude towards the BASIL intervention:

> *I feel good about taking part. It's definitely been good for me, and I hope, if this gets off the ground, that it's going to be good for other people. It was a benefit for me, it really was a big benefit to me. (OA01)*

BASIL Support Workers were positive about being part of the BASIL intervention:

> *And I want to make a difference to people, sort of, experience of healthcare and make it more positive and the best, you know, they can have. So, it felt good to be involved in something that was trying to do that (BSW03)*

Some BASIL Support Workers described positive affective attitude in terms of adapting the study in response to the COVID-19 pandemic:

"The study was adapted to BASIL to respond to COVID it felt so worthwhile and I felt really motivated to work really, really hard on it" (BSW05).

It appeared, however, that a positive affective attitude could be attenuated with study challenges, such as the difficulties with activity planning in the context of COVID-19 restrictions:

> *The constant changes in rules and the confusion that people have had over that, has, you know…it has affected it [intervention delivery]. Because one week, someone can*

For peer review only

be doing okay and the next week, rules have changed and they're feeling anxious, so they're not really sure where they stand anymore. Or, you know, restrictions have been lifted… and they've been able to go back to doing most of what they normally do, and actually, they're loads better. So you really don't know from one week to the next, where someone's going to be at. (BSW07)

Some older adult participants discussed how they had valued the BASIL Support Worker being able to help with problem-solving and information-finding:

I suppose only that if I did have a problem, there was someone there that I could discuss it with, and someone who could perhaps help me, who knew the right people to contact (OA13).

Intervention Coherence

The coherence domain of the TFA is described as *'the extent to which the participant understands the intervention, and how the intervention works'*[20]. The overall aims of the BASIL Pilot study appeared coherent to both BASIL Support Workers and older adults:

The aims are to try and help them to find ways of coping with life events such as a pandemic. But not just that. If you can teach them this behavioural activation cycle thing then they can apply it to any life event or situation that's affecting them and give them a coping strategy. (BSW08)

I presume, or this is how I looked at it, was that you were trying to get information on how people were reacting within the COVID environment, and that you were hoping to get strategies out of it, that would then help people to cope better with the situation. (OA12)

BASIL Support Workers described the ease with which they could make links between activity and low mood, which underpins the Behavioural Activation part of the BASIL intervention. BSW09 articulates how they think the intervention works, which also informs the 'perceived effectiveness' TFA domain:

For peer review only - http://bmjopen.bmj.com/site/about/guidelines.xhtml

So I think it's [BASIL intervention] set up as a structured guided support really, so that they'll get regular contact with one of the support workers who will look to help them to engage with the booklet, and maybe look at how they can make changes to what they're currently doing, in the hope that that's going to impact how they feel physically in terms of their health, but also emotionally (BSW09)

Older adults appeared to have a good understanding of the relationship between activity, mood and physical health, suggesting that the intervention was understandable to participants and had a reasonable level of face validity:

I think they link greatly because obviously if you're doing something that keeps your…makes you feel good and keeps your mind away from anxieties then that's obviously going to help you feel better, it's going to help you face each day in a better way. (OA09)

However, it appeared that the coherence of the intervention could be reduced when using Behavioural Activation with participants who were not experiencing low mood:

Some [older adults] just said because they didn't have a low mood it didn't affect their behaviour. It was very difficult to try and make them see the cycle when they didn't see any relevance for them because they were fine. (BSW08)

BASIL Support workers reflected that when people did not perceive themselves as having low mood, the intervention made less sense to them and they did not see how Behavioural Activation could work for them.

Opportunity Cost

Opportunity cost describes the '*extent to which benefits, profits, or values must be given up to engage in an intervention*'[20]. Most BASIL Support Workers talked about how their usual working role had been adjusted to accommodate the delivery of the intervention. As such

For peer review only - http://bmjopen.bmj.com/site/about/guidelines.xhtml

intervention delivery had little cost to their usual working role:

P: Yeah, my caseload was adjusted slightly, so I had a couple of client contacts less my caseload for the week to create the time to be able to work on the study.
I: Did that work okay?
P: Yeah, it was absolutely fine, I was really supported by my [work] to be able to do that, they've been brilliant. (BSW04)

Other BASIL Support Workers described how intervention delivery fitted in with their working routine: *I just fitted it in with my diary and that worked fine (BSW07).*

Generally older adults described a low opportunity cost in terms of taking part in the intervention. This was attributed, in part, to having time available: *It wasn't bothering my timetable or anything like that, cause with the lockdown I've been doing very little anyway (OA11).* Other older adults described how the intervention could be planned flexibly around their other activities:

I did, just work it in and around my life. If there was something that had to be wrote down, then I would sort of spend perhaps an hour thinking about it, and then I'd go on and do something else, and then I'd come back to it, and spend another hour maybe writing down what I wanted to say and that, you know (OA12)

<u>Burden</u>

The TFA domain of Burden describes 'The perceived amount of effort that is required to participate in the intervention'[20]. Some BASIL Support Workers described a time burden associated with delivering the intervention sessions:

If somebody lives on their own and the social isolation is more of a problem, then it's really hard to keep it to 30 minutes and generally they take an hour, those calls. (BSW06).

One BASIL Support Worker discussed the emotional effort involved in delivering the intervention in the pandemic, due to the social restrictions that everyone faced, and reported that it could be an effort to stay positive about the future:

For peer review only - http://bmjopen.bmj.com/site/about/guidelines.xhtml

It was just everything was just a bit more of an effort but that was the world per se really. It was for everybody and anybody that was working everything was more difficult, yeah. (BSW01)

Some older adults felt the amount of effort required to take part in the BASIL study was minimal, whilst others described the mental effort involved in taking part in the intervention. The data extracts below illustrate these contrasting views:

There was a little bit of effort. I mean obviously from week to week you had these tasks, your diary to fill in and to think about the tasks that you were doing that weren't enjoyable and how I could break it down into smaller chunks rather than do the whole thing in one go…So there were some things to do but it wasn't onerous at all. (OA03)

No physical effort whatsoever. Mental effort is the problem. And just, I didn't want to let the [person] who was doing the questioning, down, so I used to have to think about what was required, you know, and what [they] was expecting from me, and then try and adapt it into it. So, yes, there was a bit of effort involved, from the point of view that you don't want to do it, but you have to do it, to keep things on an even track, you know? (OA12)

Self-efficacy

The Self-efficacy domain of the TFA describes '*The participant's confidence that they can perform the behaviour(s) required to participate in the intervention*'[20]. Here, Self-efficacy is described in terms of how confident both BASIL Support Workers and older adults felt undertaking the BASIL intervention overall.

Some BASIL Support Workers described how, over time, their confidence to deliver the intervention grew with experience:

I think your confidence definitely grows with familiarity, so the first few times you're having a session with a participant, you're very keen to make sure you've got

For peer review only - http://bmjopen.bmj.com/site/about/guidelines.xhtml

everything, all your materials to hand, that you know what you're doing and what's expected of you, making sure that you're following the protocol. As it all starts to become more familiar and you can really enjoy using the booklet, it's really nice to have such nice materials to use with the client and the participant. (BSW04)

Some participants expressed a degree of increased confidence, satisfaction and mastery in having completed the intervention:

I wouldn't say confident, but I did learn a lot, and I think at the end of it, I did feel more confident. At the end of it all, I thought, well, I've done that and I'm much better off for doing it. (OA01)

Others were, however, more equivocal about engaging with the intervention but still recognised that they had become more certain about coping with the pressures of social isolation:

I didn't feel unconfident. I felt apprehensive, maybe, you know, it's all this…is this really Big Brother keeping their eye on us, blah blah blah. But I think everybody I've spoken to has been really friendly and helpful and it's helped draw me out and helped me see things better and cope with things better. No, I don't think I felt unconfident, just more apprehensive (OA14)

<u>Perceived effectiveness:</u>

Perceived effectiveness describes *'The extent to which the intervention is perceived as likely to achieve its purpose'*[20]. During the interviews, we asked if participants thought the BASIL study would achieve its aims, and to gauge how positive they felt about this, we also asked if participants would '*recommend the study to others*'[24].

Older adults reflected that the BASIL study would achieve its aims:

It does cover quite a lot really. As I say, it seems to be, you know, centred obviously towards mood and how the…this pandemic has affected how people feel and there, sort of, fears and what have you. So yes, I think it's very good (OA09)

For peer review only - http://bmjopen.bmj.com/site/about/guidelines.xhtml

BASIL Support Workers also acknowledged that Behavioural Activation had the potential to support people who were shielding and isolated during the pandemic:

> *I hadn't thought about it, in terms of delivering for COVID. And I think, yeah, it's a really good approach for this. Obviously, I've never thought about it before, I've not been in a global pandemic before, but yeah, I think it's a useful intervention. (BSW07)*

Both older adults and BASIL Support Workers reflected that the effectiveness of the BASIL intervention was likely to be enhanced if it was more specifically targeted at those with low mood and long-term physical health problems:

> *I think possibly it needs to be more targeted, so anybody who has painful medical condition or who lives alone who is isolated, certainly I think it would benefit them a lot. I think the wide spread that you've currently can be more targeted and more focused and more helpful to more people in that sense. (OA02)*

> *I think, yes, but I do think just some consideration needs to be given to who we're targeting, maybe it's not quite so useful for people on the threshold of depression and feel that they're doing quite well………..... I wonder if perhaps it would be more beneficial just to focus on people who are expressing their own thoughts of feeling low. (BSW04)*

Most participants, (Support Workers and older adults), said they would recommend the BASIL study to others:

> *Yes, definitely. But either I'd like to think that perhaps an older family member who was struggling and there was an opportunity, who was struggling who knew there was an opportunity to participate in the BASIL study, I would say yes. I'd also say yes to my colleagues because I think it really helps. (BSW04)*

> *I think, well, I'd definitely recommend it to people who want to gain some experience with working with older adults, because it's great insight. And then for the older adults*

For peer review only

For peer review only - http://bmjopen.bmj.com/site/about/guidelines.xhtml

themselves, yeah, particularly those who are struggling with low mood, I can see that it is a really valuable resource and it's helpful to have that kind of contact, even if it just gets them talking about how they're feeling, because that might not be something that they've done previously. (BSW09)

Ethicality

The Ethicality sub-theme describes '*The extent to which the intervention has a good fit with an individual's value system*'[20]. Ethicality was explored by enquiring what participants valued most or least about the BASIL intervention. BASIL Support Workers often described how they had valued being able to help older people during the pandemic:

I think most valid was just getting to know and help older adults, particularly at this time where we know mental health, particularly things like depression and things like that, has risen. So that's been [inaudible 53:15], I just like the fact that I was able to help this group of people even if it is only [number] of them I've managed to help. (BSW 06)

Other older adults described feeling positive about the BASIL intervention, primarily because it presented an opportunity to be altruistic:

I: How do you feel about taking part in the BASIL Support Package?
P: I think if it's helping the likes of yourself to understand how people are managing that if I've been able to be a part of that and assist in any way I'm pleased to have done it.(OA07)

One older adult appeared to value the social contact the intervention provided:

I think, it's actually nice to speak to people that are doing this because I think it's…in the present situation, I think anybody who's doing something like you are doing, I think is pretty good, I think it's wonderful and it's great that you're doing it, you know, for whatever reason (OA09)

Others discussed personal gains made from taking part in the intervention overall:

For peer review only - http://bmjopen.bmj.com/site/about/guidelines.xhtml

The thing I valued most is that it has made me more reflective and more grateful and that's the thing that I got out of it that it made me really look at my life and re-think how I do things (OA02)

Discussion

The BASIL Pilot study was designed to test the feasibility and acceptability of recruiting and remotely delivering a Behavioural Activation intervention to older adults with two or more long-term conditions during the COVID-19 pandemic. The intervention was set within a collaborative care framework and was designed to prevent and reduce depression and loneliness in socially isolated older adults. The initial thematic analysis (TA) (Appendix 1) suggested that the recruitment procedures to the BASIL pilot trial and the intervention (including the self-help booklet) were acceptable. Findings from the TA[21] informed the protocol for the definitive BASIL+ randomised controlled trial.[25]. Remote delivery of the intervention was acceptable and the self-help booklet was engaging and relevant, but less so for those without low mood. Activity planning during the intervention was difficult whilst COVID-19 restrictions were in place. Whilst altruism facilitated study participation, the BASIL trial needs to be targeted to those with depressive symptoms. TA highlighted detail surrounding the intervention context; variability in how the restrictions had impacted participants' mental health and perceived lack of access to primary care which could influence the way participants felt about the intervention but were not picked up by the TFA.

The TFA[20] added to the TA analysis and provided a comprehensive framework to inform the retrospective and prospective acceptability of the intervention. All seven of the TFA component constructs were populated with data from the interview transcripts and provided insights that were not identified by the initial TA. However, the ethicality component was more difficult to code, and sometimes overlapped with the Affective Attitude component. Confidence in the intervention (Self-Efficacy) grew over time for both older adults and BASIL Support Workers. For older adults, previous experience of similar interventions promoted self-efficacy, and some older adults reported greater self-efficacy as they experience positive effects from taking part. For BASIL Support Workers, self-efficacy grew with experience of delivering the intervention, although was reduced by having a long gap between training and intervention delivery. Both older adults and Support Workers valued the opportunity to demonstrate altruism in the context of the pandemic, but older adults also

For peer review only - http://bmjopen.bmj.com/site/about/guidelines.xhtml

valued the social contact and assistance with problem solving and seeking healthcare provision. The intervention had low opportunity cost for both Support Workers and older adults, who found delivering and taking part in the intervention fitted in around other commitments.

Theoretical Constraints of TFA

Burden and opportunity cost were the least populated domains – it could be that low perceived burden and low opportunity cost means participants elaborate less on these topics, or it could be that the volume and richness of the data coded to other domains indicates that those elements appealed more to participants compared to the burden or opportunity cost of the BASIL intervention. Ethicality was a difficult component to evaluate and was closely related to Affective Attitude. The questions "Is there anything you valued about the BASIL study intervention?" and "Would you recommend the BASIL study to others?" were used to target Ethicality, but it was sometimes difficult to separate participants talking about what they valued versus talking about their value system; often participants described liking the intervention for what it can offer and the benefits they gained, and it was left to the research team to interpret whether this was in line with their value system or an expression of how they felt. Perhaps Ethicality is easier to analyse in interviews with healthcare professionals with a clear set of professional values, compared to interviews with participants where their individual value system and core personal values are more implicit. Future iterations of the TFA should consider elaborating on the construct meanings and perhaps provide example questions that researchers can adopt in topic guides. The TFA approach has added value, justification for concluding that the intervention is acceptable, and provided insights into how to refine the recruitment processes for a definitive main BASIL trial (BASIL+ ISRCTN63034289) and is comprehensive enough to evaluate acceptability alone if time and resources are limited, however, carrying out an inductive TA first elucidated important contextual information that would have otherwise been missed, therefore we would recommend doing both.

Strengths & Limitations

This study was conducted as an Urgent Public Health study during the pandemic, the intervention and data collection was adapted in line with government restrictions. The use of thematic analysis followed by TFA informed the topic guide and strengthened our findings,

For peer review only - http://bmjopen.bmj.com/site/about/guidelines.xhtml

demonstrating a systematic enquiry into acceptability, and contributing to the wider field as well as the topic area. A limitation is that conducting telephone interviews may have led to a self-selecting sample of people who were comfortable with the telephone. A low intervention dropout rate of 2% in the BASIL pilot study[15] meant that there were low numbers of non-completers available to include in the qualitative study. A higher dropout rate would have enabled a larger sample of participants who did not complete the intervention. Telephone delivery of the intervention was acceptable, although some older adults suggested that if it were not for the pandemic, they would have preferred face-to-face delivery. This finding fits with previous work in the acceptability of telephone-based therapy for depression. Despite user ambivalence, there is no clear evidence that the use of the telephone negatively affects the interactional elements of therapy.[26] Furthermore, telephone-delivered case management has proven efficacy in supporting the implementation of multidisciplinary approaches to managing depression, such as collaborative care.[27] our findings offer further support for the utility of using the telephone to deliver low-intensity psychological interventions, even where there might be an expressed preference for face-to-face approaches.

To conclude, this nested qualitative study explored the perspectives and experiences of those involved in delivering and participating in the BASIL pilot study.[15] Analysis using the TFA[20] provides a novel analytic lens to understand key aspects of acceptability, but an initial TA is valuable. To our knowledge this is the first process evaluation of a trial and intervention designed to mitigate the psychological impact of COVID-19, addressing the research priority of evaluating brief psychosocial interventions to prevent depression and loneliness in vulnerable populations during the COVID-19 pandemic.[28] This contributes to the evidence around psychosocial and behavioural interventions for older adults addressing the mental health impact of COVID-19 and beyond.

For peer review only

Contributorship statement

LS and CS contributed to the writing of the manuscript. CS contributed to data acquisition, CS and AH contributed to data curation, CS, CCG, and PC contributed to data analysis. LS, CCG, PC, EN, and CS contributed to the creation and presentation of the data. EL, SGi and DE contributed to data validation. LS, CCG, PC, EN, RW and CS contributed to the editing and critical review of the manuscript. LS, CCG, PC, EN, RW, CS, AH, SG, DE, DM, ER, LB, SGa and DB contributed to the planning, conduct, and review preparation. CCG, PC, CS, EL and SGa contributed to the conceptualisation of the overarching research aims. SG, DE, CCG, EL, PC, DM contributed to funding acquisition. CCG, PC, CS, EL, SGa, LB, ER, AH, LS, DB and DM contributed to methodological design and development. CS, EL and CCG contributed to project administration, CCG, DE, SG, EL, CS contributed to study oversight and leadership.

Competing interest statement

We have read the journal's policy and the authors of this manuscript have the following competing interests: DE and CCG are current committee members for the NICE Depression Guideline (update) Development Group, and SGi was a member between 2015-18. SGi, PC and DMcM are supported by the NIHR Yorkshire and Humberside Applied Research Collaboration (ARC) and DE is supported by the North East and North Cumbria ARCs.

License statement

I, the Submitting Author has the right to grant and does grant on behalf of all authors of the Work (as defined in the below author licence), an exclusive licence and/or a non-exclusive licence for contributions from authors who are: i) UK Crown employees; ii) where BMJ has agreed a CC-BY licence shall apply, and/or iii) in accordance with the terms applicable for US Federal Government officers or employees acting as part of their official duties; on a worldwide, perpetual, irrevocable, royalty-free basis to BMJ Publishing Group Ltd ("BMJ") its licensees and where the relevant Journal is co-owned by BMJ to the co-owners of the Journal, to publish the Work in BMJ Open and any other BMJ products and to exploit all rights, as set out in our licence.

The Submitting Author accepts and understands that any supply made under these terms is made by BMJ to the Submitting Author unless you are acting as an employee on behalf of your employer or a postgraduate student of an affiliated institution which is paying any applicable article publishing charge ("APC") for Open Access articles. Where the Submitting Author wishes to make the Work available on an Open Access basis (and intends to pay the

For peer review only

For peer review only - http://bmjopen.bmj.com/site/about/guidelines.xhtml

relevant APC), the terms of reuse of such Open Access shall be governed by a Creative Commons licence – details of these licences and which Creative Commons licence will apply to this Work are set out in our licence referred to above.

Funding

This project is funded by the National Institute for Health and Care Research (NIHR) under its Programme Grants for Applied Research (PGfAR) (RP-PG-0217-20006). The funder had no role in study design, data collection and analysis, decision to publish, or preparation of the manuscript. The views expressed are those of the author(s) and not necessarily those of the NIHR or the Department of Health and Social Care.

Data sharing statement

All data relevant to the study are included in the article or uploaded as supplementary information

Ethics statement

This study involves human participants and ethical approval was provided by Yorkshire and The Humber - Leeds West Research Ethics Committee ref: 18/YH/0380 (approved as substantial amendment 02 under existing NIHR IRAS249030 research programme).

Acknowledgements

We would like to thank the participants for taking part in the trial and our Patient and Public Advisory Group members for their insightful contributions and collaboration.

For peer review only - http://bmjopen.bmj.com/site/about/guidelines.xhtml

References:

1. Peplau LA. Loneliness Research: Basic Concepts and Findings. In: Sarason IG, Sarason BR, eds. Social Support: Theory, Research and Applications. Dordrecht: Springer Netherlands 1985:269-86.
2. de Jong Gierveld J, Havens B. Cross-national Comparisons of Social Isolation and Loneliness: Introduction and Overview. *Canadian Journal on Aging / La Revue canadienne du vieillissement* 2004;23(2):109-13. doi: 10.1353/cja.2004.0021 [published Online First: 2010/03/31]
3. Hawkley, L. C., Buecker, S., Kaiser, T., & Luhmann, M. (2022). Loneliness from young adulthood to old age: Explaining age differences in loneliness. *International journal of behavioral development*, *46*(1), 39-49.
4. Gale, C. R., Westbury, L., & Cooper, C. (2018). Social isolation and loneliness as risk factors for the progression of frailty: the English Longitudinal Study of Ageing. *Age and ageing*, *47*(3), 392-397.
5. Soysal, P., Veronese, N., Thompson, T., Kahl, K. G., Fernandes, B. S., Prina, A. M. & Stubbs, B. (2017). Relationship between depression and frailty in older adults: a systematic review and meta-analysis. *Ageing research reviews*, *36*, 78-87.
6. Lee, S. L., Pearce, E., Ajnakina, O., Johnson, S., Lewis, G., Mann, F., ... & Lewis, G. (2021). The association between loneliness and depressive symptoms among adults aged 50 years and older: a 12-year population-based cohort study. *The Lancet Psychiatry*, *8*(1), 48-57.
7. Eng PM, Rimm EB, Fitzmaurice G, et al. Social ties and change in social ties in relation to subsequent total and cause-specific mortality and coronary heart disease incidence in men. *Am J Epidemiol* 2002;155(8):700-9. doi: 10.1093/aje/155.8.700 [published Online First: 2002/04/12]
8. Shankar A, Hamer M, McMunn A, et al. Social isolation and loneliness: relationships with cognitive function during 4 years of follow-up in the English Longitudinal Study of Ageing. *Psychosom Med* 2013;75(2):161-70. doi: 10.1097/PSY.0b013e31827f09cd [published Online First: 2013/01/31]
9. Dreyer K, Steventon A, Fisher R, et al. The association between living alone and health care utilisation in older adults: a retrospective cohort study of electronic health records from a London general practice. *BMC Geriatrics* 2018;18(1):269. doi: 10.1186/s12877-018-0939-4
10. Office for National Statistics. Living longer: how our population is changing and why it matters. Overview of population ageing in the UK and some of the implications for the economy, public services, society and the individual, 2018.
11. Age UK (2019) Later Life in the United Kingdom 2019. *Age UK.* https://www.ageuk.org.uk/globalassets/age-uk/documents/reports-and-publications/later_life_uk_factsheet.pdf
12. Independent Age (2021) The Mental Health Experiences of Older People During the Pandemic. *Mental Health Foundation and Independent Age.* Accessed via: https://www.independentage.org/mental-health-experiences-of-older-people-during-pandemic
13. Raina, P., Wolfson, C., Griffith, L., Kirkland, S., McMillan, J., Basta, N. & Thompson, M. (2021). A longitudinal analysis of the impact of the COVID-19 pandemic on the mental health of middle-aged and older adults from the Canadian Longitudinal Study on Aging. *Nature Aging*, *1*(12), 1137-1147.
14. HM Government. A connected society. A strategy for tackling loneliness – laying the foundations for change, 2018.

For peer review only - http://bmjopen.bmj.com/site/about/guidelines.xhtml

15. Gilbody S, Littlewood E, McMillan D, Chew-Graham CA, Bailey D, et al. (2021) Behavioural activation to prevent depression and loneliness among socially isolated older people with long-term conditions: The BASIL COVID-19 pilot randomised controlled trial. PLOS Medicine 18(10)
16. NIHR (2022) Study detail: BASIL-C19. Accessed via: https://www.nihr.ac.uk/covid-studies/study-detail.htm?entryId=249030
17. Ekers D, Webster L, Van Straten A, Cuijpers P, Richards D, et al. (2014) Behavioural Activation for Depression; An Update of Meta-Analysis of Effectiveness and Sub Group Analysis. PLOS ONE 9(6).
18. Richards, D. A., Ekers, D., McMillan, D., Taylor, R. S., Byford, S., Warren, F. C. & Finning, K. (2016). Cost and Outcome of Behavioural Activation versus Cognitive Behavioural Therapy for Depression (COBRA): a randomised, controlled, non-inferiority trial. *The Lancet*, *388*(10047), 871-880.
19. Littlewood, E., McMillan, D., Graham, C. C., Bailey, D., Gascoyne, S., Sloane, C. & Gilbody, S. (2022). Can we mitigate the psychological impacts of social isolation using behavioural activation? Long-term results of the UK BASIL Urgent Public Health COVID-19 pilot randomised controlled trial and living systematic review. *Evidence-Based Mental Health*.
20. Sekhon, M., Cartwright, M. & Francis, J.J. (2017) Acceptability of healthcare interventions: an overview of reviews and development of a theoretical framework. *BMC Health Service Research* 17, 88
21. Braun, V., & Clarke, V. (2006). Using thematic analysis in psychology. *Qualitative Research in Psychology, 3*(2), 77–101. https://doi.org/10.1191/1478088706qp063oa
22. Glaser, B. G. (1965). The constant comparative method of qualitative analysis. Social problems, 12(4), 436-445.
23. Guest, G., Bunce, A., & Johnson, L. (2006). How many interviews are enough? An experiment with data saturation and variability. Field methods, 18(1), 59-82.
24. Marshall et al (2015). The HELPER programme: HEalthy Living and Prevention of Early Relapse – three exploratory randomised controlled trials of phase-specific interventions in first-episode psychosis. Appendix 5. The InterACT trial acceptability interview topic guide. https://www.ncbi.nlm.nih.gov/books/NBK285595/
25. Burke L, Littlewood E, Gascoyne S, McMillan D, Chew-Graham CA, Bailey D, et al. (2022) Behavioural Activation for Social IsoLation (BASIL+) trial (Behavioural activation to mitigate depression and loneliness among older people with long-term conditions): Protocol for a fully-powered pragmatic randomised controlled trial. PLoS ONE 17(3).
26. Irvine A, Drew P, Bower P, et al. Are there interactional differences between telephone and face-to-face psychological therapy? A systematic review of comparative studies. *Journal of Affective Disorders* 2020;265:120-31. doi: https://doi.org/10.1016/j.jad.2020.01.057
27. Hudson JL, Bower P, Kontopantelis E, et al. Impact of telephone delivered case-management on the effectiveness of collaborative care for depression and anti-depressant use: A systematic review and meta-regression. *PLOS ONE* 2019;14(6):e0217948. doi: 10.1371/journal.pone.0217948
28. Holmes, EA., O'Connor, RC., Perry, VH. et al. (2020) Multidisciplinary research priorities for the COVID-19 pandemic: a call for action for mental health science. Lancet Psychiatry. 7: p547-560.

For peer review only - http://bmjopen.bmj.com/site/about/guidelines.xhtml

Appendix 1 Themes from thematic analysis (TA)

Theme	Descriptive summary of theme	Illustrative data extracts
Intervention context	The COVID-19 restrictions applied variably to older adults in the BASIL study (e.g., shielding, social distancing) dependent on their health status. There was considerable variability in how the restrictions had impacted participants' mental health.	*I feel as though I'm held in, I'm being persecuted because I was shielding originally. I did everything that was asked of me, we're now moving out but all of a sudden because of political decisions, I'm now finding myself back where I was. Staying in a lot, don't meet a lot of people at the best of times, generally speaking you'll probably say loneliness, isolation. (OA06)*<br><br>*But I just didn't feel motivated to do anything 'cause all the things that I really had been enjoying had just suddenly stopped overnight, literally*. (OA14)<br><br>*It's just a worry, obviously I don't want to catch it, you know, because of my underlying conditions I think I would have a rough time of it, you know …So, I'm quite happy to isolate myself, if you like (OA12)*<br><br>*The constant changes in rules and the confusion that people have had over that, has, you know…it has affected it [intervention delivery]. Because one week, someone can be doing okay and the next week, rules have changed and they're feeling anxious, so they're not really sure where they stand anymore. Or, you know, restrictions have been lifted. And so one week, they're really struggling and the next week they've been able to*

For peer review only

		go back to doing most of what they normally do, and actually, they're loads better. So it changes week to week. So you really don't know from one week to the next, where someone's going to be at, dependent on, you know, the restrictions in their area. (BSW07)
	Remote consulting / perceived lack of access to primary care	*My diabetes, I think, they've forgot about me, I have no idea I haven't…the last two appointments were supposed to be phone ones and they didn't mat…they were cancelled for different reasons. My cancer ones, I don't know what's happening there, I've missed the last two because of the same problem. They've rang up to say that either the person's not available or the computer's broke down, or…so that's my cancer and my diabetes. (OA11)*<br><br>*There's been these two sides of things. There's one side, it's not affected their long-term conditions or their access in the support. The other side is where they're not wanting to access the support because they're either too scared or they don't want to go out, and then that's having the impact on their long-term conditions 'cause they're not being able to go to their routine appointments for it and things like that. (BSW06)*
Study entry	Phased approach to recruitment (i.e.: 1) initial text from GP surgery, 2) letter with study participant information (sent via GP surgery), 3) phone call from the Clinical Research	*So, I was really quite down in the dumps a bit. And then I got this text from my doctor saying that I should take part in…[they] thought it would be beneficial for me to take part. So, I said yes, I would do that and I rang the number and said yes I would.* (OA01)

For peer review only

	Network and 4) phone call from the University study team), was both feasible and acceptable. It seemed to provide successively layered opportunities for participants to gain sufficient information and decide if they wanted to participate in the BASIL study.<br><br>Need for targeted recruitment.	*No, I mean the doctor gives you a warning that this is about to happen and then you're prepared when somebody phones up that it's not a scam, a con, which is what I don't worry about it because I know how to deal with it but to some people it could be worrying. (OANC17)*<br><br>*I think it's maybe thinking about who we would want to target in the main study. I think as I say, the participants I was working with didn't consider themselves to be struggling with their mood, they'd come into the study for altruistic reasons, rather than looking to boost their own mood and wellbeing. I guess maybe to be more targeted. (BSW04)*
Intervention delivery and content	The BASIL pilot intervention was solely delivered by telephone to older adults in their homes. Online intervention delivery was offered; however, this option was not taken up by any participants.<br><br>Duration of intervention	*I: receiving the support package over the telephone, how was that?*<br>*P: Well, I found that the person I was speaking with showed a lot of empathy, understood where I was coming from, you know, just about being on your own for long periods of time and that sort of thing. (OA06)*<br><br>*And I thought, I don't want to be bothered with not being able to get it [online platform] on, you know, I'd rather a telephone call and then I don't have the worry of the [online platform] going wrong. (OA15)*<br><br>*So if they know they've got a telephone call on a*

For peer review only

		Wednesday morning at 11, then that will really help them to structure their week and give them something to look forward to and mark their weeks. (BSW05)<br><br>*So for those who have more severe low mood, when I start working with them, I definitely see that they have more to learn in terms of using technology. (BSW06)*
	Self-help booklet	*I think it was enough because we'd done the whole book and it left me, at the end, to have an action plan. So that's all there, I'd written out on the page, so I've got that to refer to. Yes, I think eight weeks was enough. (OA15)*
	BA	*My first thought was, it was in very simple, precise language so that anybody could understand it. It wasn't, you know, sort of above anybody's head if you know what I mean. I thought it was very good, very simple. And it had different examples of what people were doing and how they were overcoming their difficulties. Yes, I thought it was very good, I liked it. (OA15)*<br><br>*And there was also plenty of opportunity for me to put my two pennorth worth in, you had pages that were blank and…list your three most priority items today……. You know, that was so…so it gave you that. (OA06)*

For peer review only

		with the booklet and the [person] who I was talking with, we were able to say what is it about you that's so important that you must have this priority and that priority, and they compete, why don't you break it down. What can I do, how much can I do, how does it interwork with each other, and it's that breaking down of… catastrophic how you are, obviously, the pandemic, how does the pandemic impact you, what can you do to circumvent it, you know, that sort of thing. And that was very useful. (OA06)<br><br>*Also, probably the breaking things down into making them more manageable. That's been particularly useful for physical health. So that helped with the physical pain. (BSW06)*<br><br>*And I felt really good to tell {them] that it made me feel better, it made me feel good. The support I got from [them] was really good because things that I'd never thought about, [they] said [themselves], why don't you…you know, have you tried this? (OA01)*

For peer review only

Appendix 2 Author Contributions using the CRediT Taxonomy

**Contributor Role**	**Role Definition**	
**Conceptualization**	Ideas; formulation or evolution of overarching research goals and aims.	CCG, PC, CS, EL, SGa
**Data Curation**	Management activities to annotate (produce metadata), scrub data and maintain research data (including software code, where it is necessary for interpreting the data itself) for initial use and later reuse.	CS, AH
**Formal Analysis**	Application of statistical, mathematical, computational, or other formal techniques to analyze or synthesize study data.	CS, CCG, PC
**Funding Acquisition**	Acquisition of the financial support for the project leading to this publication.	SG, DE, CCG, EL, PC, DM
**Investigation**	Conducting a research and investigation process, specifically performing the experiments, or data/evidence collection.	CS
**Methodology**	Development or design of methodology; creation of models	CCG, PC, CS, EL, SGa, LB, ER, AH, LS, DB, DM
**Project Administration**	Management and coordination responsibility for the research activity planning and execution.	CS, EL, CCG
**Resources**	Provision of study materials, reagents, materials, patients, laboratory samples, animals, instrumentation, computing resources, or other analysis tools.	N/A
**Software**	Programming, software development; designing computer programs; implementation of the computer code and supporting algorithms; testing of existing code components.	N/A
**Supervision**	Oversight and leadership responsibility for the research activity planning and execution, including mentorship external to the core team.	CCG, DE, SG, EL, CS
**Validation**	Verification, whether as a part of the activity or separate, of the overall replication/reproducibility of	EL, SG, DE

For peer review only

Contributor Role	Role Definition	
	results/experiments and other research outputs.	
**Visualization**	Preparation, creation and/or presentation of the published work, specifically visualization/data presentation.	LS, CCG, PC, EN, CS
**Writing – Original Draft Preparation**	Creation and/or presentation of the published work, specifically writing the initial draft (including substantive translation).	LS, CS
**Writing – Review & Editing**	Preparation, creation and/or presentation of the published work by those from the original research group, specifically critical review, commentary or revision – including pre- or post-publication stages.	LS, CCG, PC, AH, EN, SG, DE, DM, CS, ER, LB, SGa, DB, RW

For peer review only

Table 1			
Consolidated criteria for reporting qualitative studies (COREQ): 32-item checklist			
Number	Item	Guide questions/description	Location in text
Domain 1: Research team and reflexivity			
Personal Characteristics			
1.	Interviewer/facilitator	Which author/s conducted the interview or focus group?<br>Claire Sloan	Line 145, page 5
2.	Credentials	What were the researcher's credentials? E.g. PhD, MD<br>PHD in Psychology (University of Leeds)	Line 145, page 5
3.	Occupation	What was their occupation at the time of the study?<br>Research Fellow	Line 145, page 5
4.	Gender	Was the researcher male or female?<br>Female	Line 145, page 5
5.	Experience and training	What experience or training did the researcher have?<br>CS has extensive experience of the typical processes involved in health research, such as gaining REC and HRA approval, designing research protocols and recruiting/ interviewing vulnerable adults. She has also facilitated focus groups, PPI meetings, and analysed both qualitative and quantitative data for both her own research and studies she has assisted with. CS has the additional experience of thesis supervision and facilitating qualitative research seminars, as well as Deaf Awareness Training.	Line 148, page 5
Relationship with participants			
6.	Relationship established	Was a relationship established prior to study commencement?<br>No, this was avoided to eliminate possible bias	Line 145-146, page 5
7.	Participant knowledge of the interviewer	What did the participants know about the researcher? e.g. personal goals, reasons for doing the research<br>Participants had little knowledge about the interviewer (CS), just that she was researcher on the study interested in hearing about their experience of the intervention	Line 146, page 5
8.	Interviewer characteristics	What characteristics were reported about the interviewer/facilitator? e.g. Bias, assumptions, reasons and interests in the research topic.<br>N/A	
Domain 2: study design			
Theoretical framework			
9.	Methodological orientation and Theory	What methodological orientation was stated to underpin the study? e.g. grounded theory, discourse analysis, ethnography, phenomenology, content analysis<br>This research was based on two methodological approaches; the first was Thematic Analysis (TA) described by Braun and Clarke (2006) and the second was the Framework Method of Analysis, Gale et. al (2013), and its interpretation by Sekhon et.al (2017)	Line 165, page 5

For peer review only

Participant selection			
10.	Sampling	How were participants selected?<br>Consecutive sampling	Line 137, page 5
11.	Method of approach	How were participants approached? e.g. face-to-face, telephone, mail, email<br>Intervention participants who consented to being invited to a qualitative interview were contacted by telephone in order to check they were still happy to take part, and to arrange the interview. Basil support workers who provided their consent to be interviewed at their training were emailed to arrange the interview.	Line 143, page 5
12.	Sample size	How many participants were in the study?<br>15 intervention participants who completed the intervention (completers), 1 participant who did not complete the intervention (non completers) and 9 BSW's were involved in this qualitative research	Line 207-214, page 7
13.	Non-participation	How many people refused to participate or dropped out? Reasons? This is reported elsewhere and cited in the text	Line 210, page 7
Setting			
14.	Setting of data collection	Where was the data collected? e.g. home, clinic, workplace<br>Interviews were conducted remotely over the telephone.	Line 155, page 5
15.	Presence of non-participants	Was anyone else present besides the participants and researchers? No, these were one-to-one interviews (its possible that some interviewees had family members present but this was difficult to assess via telephone)	Line 155, page 5
16.	Description of sample	What are the important characteristics of the sample? e.g. demographic data, date<br>Demographic data is provided in the pilot trial paper, this is stated in the text.	Line 210, page 7
Data collection			
17.	Interview guide	Were questions, prompts, guides provided by the authors? Was it pilot tested?<br>Not provided. Pilot tested with individual from patient advisory group.	Line 203, page 7<br>Line 200-201, page 7
18.	Repeat interviews	Were repeat interviews carried out? If yes, how many?<br>No	Line 217, page 7
19.	Audio/visual recording	Did the research use audio or visual recording to collect the data?<br>Yes, an encrypted dictaphone was used to collect data from telephone interviews	Line 162, page 5
20.	Field notes	Were field notes made during and/or after the interview or focus group?	Line 164, page 5
21.	Duration	What was the duration of the interviews or focus group?	Line 163-164, page 5
22.	Data saturation	Was data saturation discussed?<br>Yes - we planned to recruit no more than 15 intervention completers as we anticipated we would hit data saturation at this point. During analysis it did become apparent that this was the case especially with particular domains of the TFA such as Burden (widely reported to be low burden to intervention participants) and Opportunity costs (widely reported to not require any sacrifice on behalf of the participants).	Line 212, page 7
23.	Transcripts returned	Were transcripts returned to participants for comment and/or correction?	Line 165, page 5

For peer review only

Domain 3: analysis and findings			
Data analysis			
24.	Number of data coders	How many data coders coded the data?	Line 192-197, page 7
25.	Description of the coding tree	Did authors provide a description of the coding tree?	N/A
26.	Derivation of themes	Were themes identified in advance or derived from the data?<br>Derived from the data	Line 220, page 7
27.	Software	What software, if applicable, was used to manage the data?<br>NVivo	Line 189, page 6
28.	Participant checking	Did participants provide feedback on the findings? No	Line 197-198, page 7
Reporting			
29.	Quotations presented	Were participant quotations presented to illustrate the themes / findings? Was each quotation identified? e.g. participant number<br>Yes	Line 234 - 487
30.	Data and findings consistent	Yes Was there consistency between the data presented and the findings?	N/A
31.	Clarity of major themes	Were major themes clearly presented in the findings?	N/A
32.	Clarity of minor themes	Is there a description of diverse cases or discussion of minor themes?	N/A

For peer review only

BMJ Open

Acceptability of a behavioural intervention to mitigate the psychological impacts of COVID-19 restrictions in older people with long-term conditions: a qualitative study

Journal:	*BMJ Open*
Manuscript ID	bmjopen-2022-064694.R2
Article Type:	Original research
Date Submitted by the Author:	24-Feb-2023
Complete List of Authors:	Shearsmith, Leanne ; University of Leeds, Leeds Institute of Health Sciences<br>Coventry, Peter; University of York, Department of Health Sciences; University of York, York Environmental Sustainability Institute<br>Sloan, Claire; University of York, Department of Health Sciences<br>Henry, Andrew; University of York, Department of Health Sciences; Tees Esk and Wear Valleys NHS Foundation Trust, Research & Development<br>Newbronner, Liz; University of York<br>Littlewood, Elizabeth; University of York, Health Sciences<br>Bailey, Della; University of York, Health Sciences<br>Gascoyne, Samantha; University of York, Department of Health Sciences<br>Burke, Lauren; University of York, Department of Health Sciences<br>Ryde, Eloise; University of York, Department of Health Sciences; Tees Esk and Wear Valleys NHS Foundation Trust, Research & Development<br>Woodhouse, Rebecca; University of York<br>McMillan, Dean; University of York, Department of Health Sciences; Hull York Medical School<br>Ekers, David; University of York, Health Sciences; Tees Esk and Wear Valleys NHS Foundation Trust, Research & Development<br>Gilbody, Simon; University of York, Department of Health Sciences<br>Chew-Graham, Carolyn; Keele University, School of Medicine
**Primary Subject Heading**:	Mental health
Secondary Subject Heading:	General practice / Family practice, Geriatric medicine, Mental health, Public health
Keywords:	COVID-19, Depression & mood disorders < PSYCHIATRY, QUALITATIVE RESEARCH

SCHOLARONE™
Manuscripts

I, the Submitting Author has the right to grant and does grant on behalf of all authors of the Work (as defined in the below author licence), an exclusive licence and/or a non-exclusive licence for contributions from authors who are: i) UK Crown employees; ii) where BMJ has agreed a CC-BY licence shall apply, and/or iii) in accordance with the terms applicable for US Federal Government officers or employees acting as part of their official duties; on a worldwide, perpetual, irrevocable, royalty-free basis to BMJ Publishing Group Ltd ("BMJ") its licensees and where the relevant Journal is co-owned by BMJ to the co-owners of the Journal, to publish the Work in this journal and any other BMJ products and to exploit all rights, as set out in our licence.

The Submitting Author accepts and understands that any supply made under these terms is made by BMJ to the Submitting Author unless you are acting as an employee on behalf of your employer or a postgraduate student of an affiliated institution which is paying any applicable article publishing charge ("APC") for Open Access articles. Where the Submitting Author wishes to make the Work available on an Open Access basis (and intends to pay the relevant APC), the terms of reuse of such Open Access shall be governed by a Creative Commons licence – details of these licences and which Creative Commons licence will apply to this Work are set out in our licence referred to above.

Other than as permitted in any relevant BMJ Author's Self Archiving Policies, I confirm this Work has not been accepted for publication elsewhere, is not being considered for publication elsewhere and does not duplicate material already published. I confirm all authors consent to publication of this Work and authorise the granting of this licence.

For peer review only

Acceptability of a behavioural intervention to mitigate the psychological impacts of COVID-19 restrictions in older people with long-term conditions: a qualitative study

Leanne Shearsmith[1*], Peter A. Coventry[2], Claire Sloan[2], Andrew Henry[2,3], Elizabeth Newbronner[2], Elizabeth Littlewood[2], Della Bailey[2], Samantha Gascoyne[2], Lauren Burke[2], Eloise Ryde[2,3], Rebecca Woodhouse[2], Dean McMillan[2,4], David Ekers[2,3], Simon Gilbody[2,4], Carolyn A. Chew-Graham[5].

* Corresponding author: Leanne Shearsmith, Leeds Institute of Health Sciences, Room 10.38A Level 10, Worsley Building, University of Leeds, LS2 9NL. Email: l.shearsmith@leeds.ac.uk

1. Leeds Institute of Health Sciences, University of Leeds, Leeds, LS2 9NL, UK
2. Department of Health Sciences, University of York, York, YO10 5DD, UK
3. Tees, Esk and Wear Valleys NHS FT, Research & Development, Flatts Lane Centre, Middlesbrough, TS6 0SZ, UK
4. Hull York Medical School, University of York, York, YO10 5DD, UK
5. School of Medicine, Keele University, Staffordshire, ST5 5BG, UK

Keywords: COVID-19, Psychosocial Intervention, Qualitative Research, Behavior Therapy, Social Isolation

Word count: 5084

For peer review only - http://bmjopen.bmj.com/site/about/guidelines.xhtml

Abstract

Objectives

The COVID-19 pandemic heightened the need to address loneliness, social isolation, and associated incidence of depression amongst older adults. Between June-October 2020, the Behavioural Activation in Social IsoLation (BASIL) pilot study investigated the acceptability and feasibility of a remotely-delivered brief psychological intervention (Behavioural Activation) to prevent and reduce loneliness and depression in older people with long-term conditions during the COVID-19 pandemic.

Design

An embedded qualitative study was conducted. Semi-structured interviews generated data that was analysed inductively using thematic analysis and then deductively using the Theoretical Framework of Acceptability (TFA).

Setting

NHS and third sector organisations in England.

Participants

Sixteen older adults and 9 support workers participating in the BASIL pilot study.

Results

Acceptability of the intervention was high across all constructs of the TFA: Older adults and BSWs support workers described a positive Affective Attitude towards the intervention linked to altruism, however the activity planning aspect of the intervention was limited due to COVID-19 restrictions. A manageable Burden was involved with delivering and participating in the intervention. For Ethicality, older adults valued social contact and making changes, support workers valued being able to observe those changes. The intervention was understood by older adults and support workers, although less understanding in older adults without low mood (Intervention Coherence). Opportunity Cost was low for support workers and older adults. Behavioural Activation was perceived to be useful in the pandemic and likely to achieve its aims (Perceived Effectiveness), especially if tailored to people with both low mood and long-term conditions. Self-efficacy developed over time and with experience for both support workers and older adults.

For peer review only - http://bmjopen.bmj.com/site/about/guidelines.xhtml

For peer review only

Conclusions

Overall, BASIL pilot study processes and the intervention were acceptable. Use of the TFA provided valuable insights into how the intervention was experienced and how the acceptability of study processes and the intervention could be enhanced ahead of the larger definitive trial (BASIL+).

Strengths & Limitations

1. The use of TFA in both informing the topic guide and conducting the analysis, demonstrating a systematic enquiry into acceptability, and contributing to the wider field as well as the topic area.
2. The length of the interviews facilitated an in-depth exploration of older adults and support workers' experiences.
3. Conducting the interviews by telephone whilst discussing feasibility of telephone delivery may have enabled contextual cues to be discussed that may have been missed in a face-to-face interview set up, however may have led to a self-selecting sample of people who were comfortable with the telephone.
4. A limitation is that the short timescale for the study meant that participants had to be interviewed as they completed 3-month outcome measures, rather than using strategic sampling.

For peer review only

Acceptability of a behavioural intervention to mitigate the psychological impacts of COVID-19 restrictions in older people with long-term conditions: a qualitative study

Introduction

Loneliness is the subjective psychological expression of social isolation owing to dissatisfaction with the frequency and quality of social contacts.[1] Social isolation is a quantifiable sense of reduced social network size and impoverished social contact.[2] Loneliness is an increasing problem across all age groups, including younger people.[3] However, loneliness is especially problematic in older adults because it is a risk factor for frailty,[4] which in turn, is a risk factor for depression.[5] The link between loneliness and subsequent depression can persist for up to 12 years after the loneliness is reported.[6] Social isolation and loneliness are also established risk factors among older adults for cognitive impairment and mortality.[7,8] In addition to the toll on the individual, loneliness impacts on health and social care services.[9]

In England the number of people aged ≥65 years is projected to grow by 20% over the next decade, and by nearly 60% in 25 years.[10] One in four older people in the UK live with a mental health condition, most commonly depression, and the prevalence is higher among older people with multi-morbidities.[11] The COVID-19 pandemic and the associated loneliness and lack of social contact has impacted the mental health of older adults.[12] Longitudinal data suggests that loneliness was an important predictor of increase in depressive symptoms in older adults during the pandemic, and that the negative mental health impacts of the pandemic persist and may worsen over the long term in the absence of effective interventions.[13] The government's strategy on loneliness sets reducing social isolation and improving well-being among older adults as a public health priority.[14]

The Behavioural Activation in Social IsoLation (BASIL) study investigated the acceptability and feasibility of a remotely delivered brief psychological intervention (Behavioural Activation) to prevent or reduce depression and loneliness in older people with long term conditions during isolation (or shielding).[15] The study was adopted by the NIHR Urgent Public Health programme in May 2020.[16] Behavioural Activation is an evidence-based psychological treatment. It works on the principle that low mood may be a consequence of

For peer review only - http://bmjopen.bmj.com/site/about/guidelines.xhtml

physical inactivity, and loss of positive reinforcement due to a reduction in valued, pleasurable activities.[17] It is a simpler psychological treatment than Cognitive Behavioural Therapy, but evidence suggests it can be an equally effective therapy for depression, even when delivered by junior mental health workers requiring less intensive and costly training.[18] Emerging evidence suggests that behavioural activation has an impact on depression and loneliness in the short term.[19] A bespoke intervention was used in the BASIL study incorporating Behavioural Activation, a self-help booklet and trained BASIL Support Workers, with the aim of encouraging the older adult to recognise the link between mood and behaviour, and reinstate or replace valued activities that generate positive reinforcement and so improve mood.

The BASIL pilot study findings have been reported elsewhere.[15,19] This paper reports the findings from the qualitative study within BASIL, presenting analysis using the Theoretical Framework of Acceptability (TFA).[20]

Methods

We conducted a nested qualitative study, using semi-structured interviews to explore the acceptability and feasibility of the intervention from the perspectives of older adult participants and BASIL Support Workers.

Ethical approval was provided by Yorkshire and The Humber - Leeds West Research Ethics Committee ref: 18/YH/0380 (approved as substantial amendment 02 under existing NIHR IRAS249030 research programme). The protocol for the BASIL pilot study was preregistered (ISRCTN94091479) on June 9, 2020.

Intervention

Behavioural activation was adapted with input from our patient and public advisory group. Support workers from a range of clinical and research backgrounds (Psychological wellbeing practitioners, mental health nurses, research assistants, clinical support officers, research nurses and crisis support workers) were trained to deliver the intervention by telephone or video call across 8 sessions. The intervention is described in more detail elsewhere.[19]

Participant Selection

Sixteen study participants were selected via consecutive sampling and interviewed from a pool of 86 study participants from the BASIL pilot study who consented to take part in an

For peer review only - http://bmjopen.bmj.com/site/about/guidelines.xhtml

interview at initial recruitment (inclusion criteria: aged 65 years or over with two or more physical long-term conditions). Two BASIL pilot study participants did not complete the intervention and one of these participants consented to interview as a non-completer. All nine BASIL Support Workers consented to participate in an interview. Study participants were approached via telephone following completion of the intervention and one-month post-randomisation follow-up measures. Participants were contacted by the researcher (CS) and interviews were arranged at a time to suit the participant. No relationship between participant and researcher was established prior to study commencement. Participants had minimal knowledge about the interviewer, and questions were not provided before the interview. Interviews were conducted by CS, a female research fellow with a PhD in Psychology. CS had extensive experience of interviewing and analysis of qualitative data, and Deaf Awareness training. BASIL Support Workers provided their verbal permission to be contacted followed completion of the BASIL intervention training. BASIL Support Workers were provided with study information and invited to contact the interviewing researcher if they were willing to participate in an interview.

Data Collection

One-to-one semi-structured interviews were conducted by telephone. The interviews took place between September and November 2020. Topic guides explored intervention delivery context (e.g., impact of the COVID-19 context on older adults), study processes (e.g. remote recruitment, mode of intervention delivery) and thoughts around intervention content (e.g., study materials). Study topic guides were sensitised by the seven constructs of the Theoretical Framework of Acceptability (TFA)[20] (Table 1).

Interviews were digitally-recorded using an encrypted dictaphone and transcribed verbatim by a professional transcribing company. The 'completer' interviews lasted between 30 and 56 minutes, and BASIL Support Worker interviews 39 and 60 minutes. Field notes were not made during or after the interview. Transcripts were not returned to participants.

Analysis

We initially carried out an iterative thematic analysis,[21] so that we were able to fully explore and familiarise ourselves with the data (reported elsewhere[15]). We then used the theoretical derived Theoretical Framework of Acceptability (TFA)[20], which clearly articulates and defines a range of conceptually distinct constructs related to acceptability of interventions

For peer review only - http://bmjopen.bmj.com/site/about/guidelines.xhtml

(Table 1). These constructs go beyond operational definitions of acceptability such as drop-out and uptake rates of interventions which are often more aligned with notions of satisfaction than acceptability. Crucially the TFA can be used to capture responses about intervention acceptability from both recipients and those involved in delivering interventions. Additionally the TFA can be used to assess different aspects of acceptability before, during, or after intervention delivery, depending on intervention development and evaluation cycle.

Table 1 Overview of TFA Domains

Component	Definition
Affective Attitude	How an individual feels about the intervention
Burden	The perceived amount of effort that is required to participate in the intervention
Ethicality	The extent to which the intervention has good fit with the individual's value system
Intervention Coherence	The extent to which the participant understands the intervention and how it works
Opportunity Costs	The extent to which benefits, profits or values must be given up to engage in the intervention
Perceived Effectiveness	The extent to which the intervention is perceived as likely to achieve its purpose
Self-efficacy	The participant's confidence that they can perform the behaviour(s) required to participate in the intervention

Table 1. The seven-component construct of the Theoretical Framework of Acceptability, adapted from Sekhon, Cartwright & Francis (2017).

Using a theoretically derived framework specifically designed for assessing the acceptability of healthcare interventions was considered appropriate for this study, alongside a thematic analysis to provide a multi-dimensional assessment of the acceptability of the intervention and pilot study processes. We used the TFA after intervention delivery to elicit responses about the anticipated acceptability of the content and delivery of the BASIL intervention with a view to informing refinement of intervention features in advance of definitive evaluation.

Familiarisation of data relevant to the TFA was undertaken by drawing on the meaning of the domains[20] and then indexing data considered relevant to these domains using NVIVO 12. This analysis was carried out using Constant Comparison[22] methods and regular research meetings were conducted to agree interpretation, relevance and meaning of indexing to the TFA domains. Data were then analysed by CS and AH within the TFA domains, initially by participant group. Constant Comparison methods were then applied to compare and contrast

For peer review only - http://bmjopen.bmj.com/site/about/guidelines.xhtml

data across participant groups (data mapping) by CS. The Constant Comparison[22] method facilitated the development of categories of phenomena within the TFA domains that allowed for interpretation of participants' perceptions of the acceptability of the BASIL study. Final data interpretation was agreed through discussion with CS, AH, CCG and PC. Participants did not provide feedback on the findings.

Patient and Public Involvement and Engagement

Stakeholder groups (with older adults, carers, clinicians and third sector practitioners) had been conducted pre-pandemic to develop the intervention and then online in April 2020 to refine the intervention for remote delivery. The public-facing documents were discussed with our patient advisory group prior to submission for ethics approval. The questions were pilot tested with an individual from the patient advisory group. The findings and analysis from the qualitative study were discussed with the patient participation group.

For peer review only

Results

Participants

Semi-structured interviews were conducted with 15 participants who had completed the intervention ('completers'), and one with a participant who did not complete the intervention ('non-completer'). Data saturation was reached with 15 completer participants,[23] who had between 2-8 of a maximum 8 intervention sessions (mean = 6.5). Participant demographics for the pilot trial are reported elsewhere[15] along with a summary of the thematic analysis (see also Appendix 1).

Nine interviews were carried out with the BASIL Support Workers who delivered the intervention (characteristics reported elsewhere[15]).

No repeat interviews were carried out.

Themes derived from the data using Thematic analysis (Intervention context, Study entry, Intervention delivery and content) are presented in Appendix 1.

Findings from the TFA analysis

This paper focuses on our findings within the Theoretical Framework of Acceptability (TFA), incorporating both older adult and BASIL Support Worker perspectives. Throughout the paper and Appendix 1, illustrative data is given with participant identifiers. Older adult

For peer review only - http://bmjopen.bmj.com/site/about/guidelines.xhtml

intervention 'completer' quotes are labelled with the prefix 'OA', then their identifier (e.g. OA09). The intervention 'non-completer' participant is labelled with the prefix 'OA', then 'NC' (NC: non-completer), before their identifier (e.g. OA NC07). BASIL Support Worker quotes are labelled with the prefix 'BSW', then their identifier (e.g., BSW03). The findings from the TFA analysis are presented under each TFA domain.

Affective Attitude

Affective attitude describes 'How an individual feels about the intervention'[20]. Affective attitude is described here in terms of the overall retrospective attitude to receiving or delivering the BASIL intervention. Older adults described a positive affective attitude towards the BASIL intervention:

> *I feel good about taking part. It's definitely been good for me, and I hope, if this gets off the ground, that it's going to be good for other people. It was a benefit for me, it really was a big benefit to me. (OA01)*

BASIL Support Workers were positive about being part of the BASIL intervention:

> *And I want to make a difference to people, sort of, experience of healthcare and make it more positive and the best, you know, they can have. So, it felt good to be involved in something that was trying to do that (BSW03)*

Some BASIL Support Workers described positive affective attitude in terms of adapting the study in response to the COVID-19 pandemic:

"The study was adapted to BASIL to respond to COVID it felt so worthwhile and I felt really motivated to work really, really hard on it" (BSW05).

It appeared, however, that a positive affective attitude could be attenuated with study challenges, such as the difficulties with activity planning in the context of COVID-19 restrictions:

> *The constant changes in rules and the confusion that people have had over that, has, you know…it has affected it [intervention delivery]. Because one week, someone can*

For peer review only - http://bmjopen.bmj.com/site/about/guidelines.xhtml

be doing okay and the next week, rules have changed and they're feeling anxious, so they're not really sure where they stand anymore. Or, you know, restrictions have been lifted… and they've been able to go back to doing most of what they normally do, and actually, they're loads better. So you really don't know from one week to the next, where someone's going to be at. (BSW07)

Some older adult participants discussed how they had valued the BASIL Support Worker being able to help with problem-solving and information-finding:

I suppose only that if I did have a problem, there was someone there that I could discuss it with, and someone who could perhaps help me, who knew the right people to contact (OA13).

<u>Intervention Coherence</u>

The coherence domain of the TFA is described as *'the extent to which the participant understands the intervention, and how the intervention works'*[20]. The overall aims of the BASIL Pilot study appeared coherent to both BASIL Support Workers and older adults:

The aims are to try and help them to find ways of coping with life events such as a pandemic. But not just that. If you can teach them this behavioural activation cycle thing then they can apply it to any life event or situation that's affecting them and give them a coping strategy. (BSW08)

I presume, or this is how I looked at it, was that you were trying to get information on how people were reacting within the COVID environment, and that you were hoping to get strategies out of it, that would then help people to cope better with the situation. (OA12)

BASIL Support Workers described the ease with which they could make links between activity and low mood, which underpins the Behavioural Activation part of the BASIL intervention. BSW09 articulates how they think the intervention works, which also informs the 'perceived effectiveness' TFA domain:

For peer review only - http://bmjopen.bmj.com/site/about/guidelines.xhtml

So I think it's [BASIL intervention] set up as a structured guided support really, so that they'll get regular contact with one of the support workers who will look to help them to engage with the booklet, and maybe look at how they can make changes to what they're currently doing, in the hope that that's going to impact how they feel physically in terms of their health, but also emotionally (BSW09)

Older adults appeared to have a good understanding of the relationship between activity, mood and physical health, suggesting that the intervention was understandable to participants and had a reasonable level of face validity:

I think they link greatly because obviously if you're doing something that keeps your…makes you feel good and keeps your mind away from anxieties then that's obviously going to help you feel better, it's going to help you face each day in a better way. (OA09)

However, it appeared that the coherence of the intervention could be reduced when using Behavioural Activation with participants who were not experiencing low mood:

Some [older adults] just said because they didn't have a low mood it didn't affect their behaviour. It was very difficult to try and make them see the cycle when they didn't see any relevance for them because they were fine. (BSW08)

BASIL Support workers reflected that when people did not perceive themselves as having low mood, the intervention made less sense to them and they did not see how Behavioural Activation could work for them.

<u>Opportunity Cost</u>

Opportunity cost describes the '*extent to which benefits, profits, or values must be given up to engage in an intervention*'[20]. Most BASIL Support Workers talked about how their usual working role had been adjusted to accommodate the delivery of the intervention. As such

For peer review only - http://bmjopen.bmj.com/site/about/guidelines.xhtml

intervention delivery had little cost to their usual working role:

> *P: Yeah, my caseload was adjusted slightly, so I had a couple of client contacts less my caseload for the week to create the time to be able to work on the study.*
> *I: Did that work okay?*
> *P: Yeah, it was absolutely fine, I was really supported by my [work] to be able to do that, they've been brilliant. (BSW04)*

Other BASIL Support Workers described how intervention delivery fitted in with their working routine: *I just fitted it in with my diary and that worked fine (BSW07).*

Generally older adults described a low opportunity cost in terms of taking part in the intervention. This was attributed, in part, to having time available: *It wasn't bothering my timetable or anything like that, cause with the lockdown I've been doing very little anyway (OA11).* Other older adults described how the intervention could be planned flexibly around their other activities:

> *I did, just work it in and around my life. If there was something that had to be wrote down, then I would sort of spend perhaps an hour thinking about it, and then I'd go on and do something else, and then I'd come back to it, and spend another hour maybe writing down what I wanted to say and that, you know (OA12)*

<u>Burden</u>

The TFA domain of Burden describes 'The perceived amount of effort that is required to participate in the intervention'[20]. Some BASIL Support Workers described a time burden associated with delivering the intervention sessions:

> *If somebody lives on their own and the social isolation is more of a problem, then it's really hard to keep it to 30 minutes and generally they take an hour, those calls. (BSW06).*

One BASIL Support Worker discussed the emotional effort involved in delivering the intervention in the pandemic, due to the social restrictions that everyone faced, and reported that it could be an effort to stay positive about the future:

For peer review only

For peer review only - http://bmjopen.bmj.com/site/about/guidelines.xhtml

> *It was just everything was just a bit more of an effort but that was the world per se really. It was for everybody and anybody that was working everything was more difficult, yeah. (BSW01)*

Some older adults felt the amount of effort required to take part in the BASIL study was minimal, whilst others described the mental effort involved in taking part in the intervention. The data extracts below illustrate these contrasting views:

> *There was a little bit of effort. I mean obviously from week to week you had these tasks, your diary to fill in and to think about the tasks that you were doing that weren't enjoyable and how I could break it down into smaller chunks rather than do the whole thing in one go…So there were some things to do but it wasn't onerous at all. (OA03)*

> *No physical effort whatsoever. Mental effort is the problem. And just, I didn't want to let the [person] who was doing the questioning, down, so I used to have to think about what was required, you know, and what [they] was expecting from me, and then try and adapt it into it. So, yes, there was a bit of effort involved, from the point of view that you don't want to do it, but you have to do it, to keep things on an even track, you know? (OA12)*

<u>Self-efficacy</u>

The Self-efficacy domain of the TFA describes '*The participant's confidence that they can perform the behaviour(s) required to participate in the intervention*'[20]. Here, Self-efficacy is described in terms of how confident both BASIL Support Workers and older adults felt undertaking the BASIL intervention overall.

Some BASIL Support Workers described how, over time, their confidence to deliver the intervention grew with experience:

> *I think your confidence definitely grows with familiarity, so the first few times you're having a session with a participant, you're very keen to make sure you've got*

For peer review only

For peer review only - http://bmjopen.bmj.com/site/about/guidelines.xhtml

everything, all your materials to hand, that you know what you're doing and what's expected of you, making sure that you're following the protocol. As it all starts to become more familiar and you can really enjoy using the booklet, it's really nice to have such nice materials to use with the client and the participant. (BSW04)

Some participants expressed a degree of increased confidence, satisfaction and mastery in having completed the intervention:

I wouldn't say confident, but I did learn a lot, and I think at the end of it, I did feel more confident. At the end of it all, I thought, well, I've done that and I'm much better off for doing it. (OA01)

Others were, however, more equivocal about engaging with the intervention but still recognised that they had become more certain about coping with the pressures of social isolation:

I didn't feel unconfident. I felt apprehensive, maybe, you know, it's all this…is this really Big Brother keeping their eye on us, blah blah blah. But I think everybody I've spoken to has been really friendly and helpful and it's helped draw me out and helped me see things better and cope with things better. No, I don't think I felt unconfident, just more apprehensive (OA14)

<u>Perceived effectiveness:</u>

Perceived effectiveness describes *'The extent to which the intervention is perceived as likely to achieve its purpose'*[20]. During the interviews, we asked if participants thought the BASIL study would achieve its aims, and to gauge how positive they felt about this, we also asked if participants would '*recommend the study to others*'[24].

Older adults reflected that the BASIL study would achieve its aims:

It does cover quite a lot really. As I say, it seems to be, you know, centred obviously towards mood and how the…this pandemic has affected how people feel and there, sort of, fears and what have you. So yes, I think it's very good (OA09)

For peer review only - http://bmjopen.bmj.com/site/about/guidelines.xhtml

BASIL Support Workers also acknowledged that Behavioural Activation had the potential to support people who were shielding and isolated during the pandemic:

I hadn't thought about it, in terms of delivering for COVID. And I think, yeah, it's a really good approach for this. Obviously, I've never thought about it before, I've not been in a global pandemic before, but yeah, I think it's a useful intervention. (BSW07)

Both older adults and BASIL Support Workers reflected that the effectiveness of the BASIL intervention was likely to be enhanced if it was more specifically targeted at those with low mood and long-term physical health problems:

I think possibly it needs to be more targeted, so anybody who has painful medical condition or who lives alone who is isolated, certainly I think it would benefit them a lot. I think the wide spread that you've currently can be more targeted and more focused and more helpful to more people in that sense. (OA02)

I think, yes, but I do think just some consideration needs to be given to who we're targeting, maybe it's not quite so useful for people on the threshold of depression and feel that they're doing quite well………..… I wonder if perhaps it would be more beneficial just to focus on people who are expressing their own thoughts of feeling low. (BSW04)

Most participants, (Support Workers and older adults), said they would recommend the BASIL study to others:

Yes, definitely. But either I'd like to think that perhaps an older family member who was struggling and there was an opportunity, who was struggling who knew there was an opportunity to participate in the BASIL study, I would say yes. I'd also say yes to my colleagues because I think it really helps. (BSW04)

I think, well, I'd definitely recommend it to people who want to gain some experience with working with older adults, because it's great insight. And then for the older adults

For peer review only - http://bmjopen.bmj.com/site/about/guidelines.xhtml

themselves, yeah, particularly those who are struggling with low mood, I can see that it is a really valuable resource and it's helpful to have that kind of contact, even if it just gets them talking about how they're feeling, because that might not be something that they've done previously. (BSW09)

Ethicality

The Ethicality sub-theme describes '*The extent to which the intervention has a good fit with an individual's value system'*[20]. Ethicality was explored by enquiring what participants valued most or least about the BASIL intervention. BASIL Support Workers often described how they had valued being able to help older people during the pandemic:

I think most valid was just getting to know and help older adults, particularly at this time where we know mental health, particularly things like depression and things like that, has risen. So that's been [inaudible 53:15], I just like the fact that I was able to help this group of people even if it is only [number] of them I've managed to help. (BSW 06)

Other older adults described feeling positive about the BASIL intervention, primarily because it presented an opportunity to be altruistic:

I: How do you feel about taking part in the BASIL Support Package?
P: I think if it's helping the likes of yourself to understand how people are managing that if I've been able to be a part of that and assist in any way I'm pleased to have done it.(OA07)

One older adult appeared to value the social contact the intervention provided:

I think, it's actually nice to speak to people that are doing this because I think it's…in the present situation, I think anybody who's doing something like you are doing, I think is pretty good, I think it's wonderful and it's great that you're doing it, you know, for whatever reason (OA09)

Others discussed personal gains made from taking part in the intervention overall:

For peer review only - http://bmjopen.bmj.com/site/about/guidelines.xhtml

The thing I valued most is that it has made me more reflective and more grateful and that's the thing that I got out of it that it made me really look at my life and re-think how I do things (OA02)

Discussion

The BASIL Pilot study was designed to test the feasibility and acceptability of recruiting and remotely delivering a Behavioural Activation intervention to older adults with two or more long-term conditions during the COVID-19 pandemic. The intervention was set within a collaborative care framework and was designed to prevent and reduce depression and loneliness in socially isolated older adults. The initial thematic analysis (TA) (Appendix 1) suggested that the recruitment procedures to the BASIL pilot trial and the intervention (including the self-help booklet) were acceptable. Findings from the TA[21] informed the protocol for the definitive BASIL+ randomised controlled trial.[25]. Remote delivery of the intervention was acceptable and the self-help booklet was engaging and relevant, but less so for those without low mood. Activity planning during the intervention was difficult whilst COVID-19 restrictions were in place. Whilst altruism facilitated study participation, the BASIL trial needs to be targeted to those with depressive symptoms. TA highlighted detail surrounding the intervention context; variability in how the restrictions had impacted participants' mental health and perceived lack of access to primary care which could influence the way participants felt about the intervention but were not picked up by the TFA.

The TFA[20] added to the TA analysis and provided a comprehensive framework to inform the retrospective and prospective acceptability of the intervention. All seven of the TFA component constructs were populated with data from the interview transcripts and provided insights that were not identified by the initial TA. However, the ethicality component was more difficult to code, and sometimes overlapped with the Affective Attitude component. Confidence in the intervention (Self-Efficacy) grew over time for both older adults and BASIL Support Workers. For older adults, previous experience of similar interventions promoted self-efficacy, and some older adults reported greater self-efficacy as they experience positive effects from taking part. For BASIL Support Workers, self-efficacy grew with experience of delivering the intervention, although was reduced by having a long gap between training and intervention delivery. Both older adults and Support Workers valued the opportunity to demonstrate altruism in the context of the pandemic, but older adults also

For peer review only - http://bmjopen.bmj.com/site/about/guidelines.xhtml

valued the social contact and assistance with problem solving and seeking healthcare provision. The intervention had low opportunity cost for both Support Workers and older adults, who found delivering and taking part in the intervention fitted in around other commitments.

Theoretical Constraints of TFA

Burden and opportunity cost were the least populated domains – it could be that low perceived burden and low opportunity cost means participants elaborate less on these topics, or it could be that the volume and richness of the data coded to other domains indicates that those elements appealed more to participants compared to the burden or opportunity cost of the BASIL intervention. Ethicality was a difficult component to evaluate and was closely related to Affective Attitude. The questions "Is there anything you valued about the BASIL study intervention?" and "Would you recommend the BASIL study to others?" were used to target Ethicality, but it was sometimes difficult to separate participants talking about what they valued versus talking about their value system; often participants described liking the intervention for what it can offer and the benefits they gained, and it was left to the research team to interpret whether this was in line with their value system or an expression of how they felt. Perhaps Ethicality is easier to analyse in interviews with healthcare professionals with a clear set of professional values, compared to interviews with participants where their individual value system and core personal values are more implicit. Future iterations of the TFA should consider elaborating on the construct meanings and perhaps provide example questions that researchers can adopt in topic guides. The TFA approach has added value, justification for concluding that the intervention is acceptable, and provided insights into how to refine the recruitment processes for a definitive main BASIL trial (BASIL+ ISRCTN63034289) and is comprehensive enough to evaluate acceptability alone if time and resources are limited, however, carrying out an inductive TA first elucidated important contextual information that would have otherwise been missed, therefore we would recommend doing both.

Strengths & Limitations

This study was conducted as an Urgent Public Health study during the pandemic, the intervention and data collection was adapted in line with government restrictions. The use of thematic analysis followed by TFA informed the topic guide and strengthened our findings,

For peer review only - http://bmjopen.bmj.com/site/about/guidelines.xhtml

demonstrating a systematic enquiry into acceptability, and contributing to the wider field as well as the topic area. A limitation is that conducting telephone interviews may have led to a self-selecting sample of people who were comfortable with the telephone. A low intervention dropout rate of 2% in the BASIL pilot study[15] meant that there were low numbers of non-completers available to include in the qualitative study. A higher dropout rate would have enabled a larger sample of participants who did not complete the intervention. Telephone delivery of the intervention was acceptable, although some older adults suggested that if it were not for the pandemic, they would have preferred face-to-face delivery. This finding fits with previous work in the acceptability of telephone-based therapy for depression. Despite user ambivalence, there is no clear evidence that the use of the telephone negatively affects the interactional elements of therapy.[26] Furthermore, telephone-delivered case management has proven efficacy in supporting the implementation of multidisciplinary approaches to managing depression, such as collaborative care.[27] our findings offer further support for the utility of using the telephone to deliver low-intensity psychological interventions, even where there might be an expressed preference for face-to-face approaches.

To conclude, this nested qualitative study explored the perspectives and experiences of those involved in delivering and participating in the BASIL pilot study.[15] Analysis using the TFA[20] provides a novel analytic lens to understand key aspects of acceptability, but an initial TA is valuable. To our knowledge this is the first process evaluation of a trial and intervention designed to mitigate the psychological impact of COVID-19, addressing the research priority of evaluating brief psychosocial interventions to prevent depression and loneliness in vulnerable populations during the COVID-19 pandemic.[28] This contributes to the evidence around psychosocial and behavioural interventions for older adults addressing the mental health impact of COVID-19 and beyond.

For peer review only - http://bmjopen.bmj.com/site/about/guidelines.xhtml

Contributorship statement

LS and CS contributed to the writing of the manuscript. CS contributed to data acquisition, CS and AH contributed to data curation, CS, CCG, and PC contributed to data analysis. LS, CCG, PC, EN, and CS contributed to the creation and presentation of the data. EL, SGi and DE contributed to data validation. LS, CCG, PC, EN, RW and CS contributed to the editing and critical review of the manuscript. LS, CCG, PC, EN, RW, CS, AH, SG, DE, DM, ER, LB, SGa and DB contributed to the planning, conduct, and review preparation. CCG, PC, CS, EL and SGa contributed to the conceptualisation of the overarching research aims. SG, DE, CCG, EL, PC, DM contributed to funding acquisition. CCG, PC, CS, EL, SGa, LB, ER, AH, LS, DB and DM contributed to methodological design and development. CS, EL and CCG contributed to project administration, CCG, DE, SG, EL, CS contributed to study oversight and leadership.

Competing interest statement

We have read the journal's policy and the authors of this manuscript have the following competing interests: DE and CCG are current committee members for the NICE Depression Guideline (update) Development Group, and SGi was a member between 2015-18. SGi, PC and DMcM are supported by the NIHR Yorkshire and Humberside Applied Research Collaboration (ARC) and DE is supported by the North East and North Cumbria ARCs.

License statement

I, the Submitting Author has the right to grant and does grant on behalf of all authors of the Work (as defined in the below author licence), an exclusive licence and/or a non-exclusive licence for contributions from authors who are: i) UK Crown employees; ii) where BMJ has agreed a CC-BY licence shall apply, and/or iii) in accordance with the terms applicable for US Federal Government officers or employees acting as part of their official duties; on a worldwide, perpetual, irrevocable, royalty-free basis to BMJ Publishing Group Ltd ("BMJ") its licensees and where the relevant Journal is co-owned by BMJ to the co-owners of the Journal, to publish the Work in BMJ Open and any other BMJ products and to exploit all rights, as set out in our licence.

The Submitting Author accepts and understands that any supply made under these terms is made by BMJ to the Submitting Author unless you are acting as an employee on behalf of your employer or a postgraduate student of an affiliated institution which is paying any applicable article publishing charge ("APC") for Open Access articles. Where the Submitting Author wishes to make the Work available on an Open Access basis (and intends to pay the

For peer review only

For peer review only - http://bmjopen.bmj.com/site/about/guidelines.xhtml

relevant APC), the terms of reuse of such Open Access shall be governed by a Creative Commons licence – details of these licences and which Creative Commons licence will apply to this Work are set out in our licence referred to above.

Funding

This project is funded by the National Institute for Health and Care Research (NIHR) under its Programme Grants for Applied Research (PGfAR) (RP-PG-0217-20006). The funder had no role in study design, data collection and analysis, decision to publish, or preparation of the manuscript. The views expressed are those of the author(s) and not necessarily those of the NIHR or the Department of Health and Social Care.

Data sharing statement

All data relevant to the study are included in the article or uploaded as supplementary information

Ethics statement

This study involves human participants and ethical approval was provided by Yorkshire and The Humber - Leeds West Research Ethics Committee ref: 18/YH/0380 (approved as substantial amendment 02 under existing NIHR IRAS249030 research programme).

Acknowledgements

We would like to thank the participants for taking part in the trial and our Patient and Public Advisory Group members for their insightful contributions and collaboration.

For peer review only

References:

1. Peplau LA. Loneliness Research: Basic Concepts and Findings. In: Sarason IG, Sarason BR, eds. Social Support: Theory, Research and Applications. Dordrecht: Springer Netherlands 1985:269-86.
2. de Jong Gierveld J, Havens B. Cross-national Comparisons of Social Isolation and Loneliness: Introduction and Overview. *Canadian Journal on Aging / La Revue canadienne du vieillissement* 2004;23(2):109-13. doi: 10.1353/cja.2004.0021 [published Online First: 2010/03/31]
3. Hawkley, L. C., Buecker, S., Kaiser, T., & Luhmann, M. (2022). Loneliness from young adulthood to old age: Explaining age differences in loneliness. *International journal of behavioral development*, *46*(1), 39-49.
4. Gale, C. R., Westbury, L., & Cooper, C. (2018). Social isolation and loneliness as risk factors for the progression of frailty: the English Longitudinal Study of Ageing. *Age and ageing*, *47*(3), 392-397.
5. Soysal, P., Veronese, N., Thompson, T., Kahl, K. G., Fernandes, B. S., Prina, A. M. & Stubbs, B. (2017). Relationship between depression and frailty in older adults: a systematic review and meta-analysis. *Ageing research reviews*, *36*, 78-87.
6. Lee, S. L., Pearce, E., Ajnakina, O., Johnson, S., Lewis, G., Mann, F., ... & Lewis, G. (2021). The association between loneliness and depressive symptoms among adults aged 50 years and older: a 12-year population-based cohort study. *The Lancet Psychiatry*, *8*(1), 48-57.
7. Eng PM, Rimm EB, Fitzmaurice G, et al. Social ties and change in social ties in relation to subsequent total and cause-specific mortality and coronary heart disease incidence in men. *Am J Epidemiol* 2002;155(8):700-9. doi: 10.1093/aje/155.8.700 [published Online First: 2002/04/12]
8. Shankar A, Hamer M, McMunn A, et al. Social isolation and loneliness: relationships with cognitive function during 4 years of follow-up in the English Longitudinal Study of Ageing. *Psychosom Med* 2013;75(2):161-70. doi: 10.1097/PSY.0b013e31827f09cd [published Online First: 2013/01/31]
9. Dreyer K, Steventon A, Fisher R, et al. The association between living alone and health care utilisation in older adults: a retrospective cohort study of electronic health records from a London general practice. *BMC Geriatrics* 2018;18(1):269. doi: 10.1186/s12877-018-0939-4
10. Office for National Statistics. Living longer: how our population is changing and why it matters. Overview of population ageing in the UK and some of the implications for the economy, public services, society and the individual, 2018.
11. Age UK (2019) Later Life in the United Kingdom 2019. *Age UK.* https://www.ageuk.org.uk/globalassets/age-uk/documents/reports-and-publications/later_life_uk_factsheet.pdf
12. Independent Age (2021) The Mental Health Experiences of Older People During the Pandemic. *Mental Health Foundation and Independent Age.* Accessed via: https://www.independentage.org/mental-health-experiences-of-older-people-during-pandemic
13. Raina, P., Wolfson, C., Griffith, L., Kirkland, S., McMillan, J., Basta, N. & Thompson, M. (2021). A longitudinal analysis of the impact of the COVID-19 pandemic on the mental health of middle-aged and older adults from the Canadian Longitudinal Study on Aging. *Nature Aging*, *1*(12), 1137-1147.
14. HM Government. A connected society. A strategy for tackling loneliness – laying the foundations for change, 2018.

For peer review only - http://bmjopen.bmj.com/site/about/guidelines.xhtml

15. Gilbody S, Littlewood E, McMillan D, Chew-Graham CA, Bailey D, et al. (2021) Behavioural activation to prevent depression and loneliness among socially isolated older people with long-term conditions: The BASIL COVID-19 pilot randomised controlled trial. PLOS Medicine 18(10)
16. NIHR (2022) Study detail: BASIL-C19. Accessed via: https://www.nihr.ac.uk/covid-studies/study-detail.htm?entryId=249030
17. Ekers D, Webster L, Van Straten A, Cuijpers P, Richards D, et al. (2014) Behavioural Activation for Depression; An Update of Meta-Analysis of Effectiveness and Sub Group Analysis. PLOS ONE 9(6).
18. Richards, D. A., Ekers, D., McMillan, D., Taylor, R. S., Byford, S., Warren, F. C. & Finning, K. (2016). Cost and Outcome of Behavioural Activation versus Cognitive Behavioural Therapy for Depression (COBRA): a randomised, controlled, non-inferiority trial. *The Lancet*, *388*(10047), 871-880.
19. Littlewood, E., McMillan, D., Graham, C. C., Bailey, D., Gascoyne, S., Sloane, C. & Gilbody, S. (2022). Can we mitigate the psychological impacts of social isolation using behavioural activation? Long-term results of the UK BASIL Urgent Public Health COVID-19 pilot randomised controlled trial and living systematic review. *Evidence-Based Mental Health*.
20. Sekhon, M., Cartwright, M. & Francis, J.J. (2017) Acceptability of healthcare interventions: an overview of reviews and development of a theoretical framework. *BMC Health Service Research* 17, 88
21. Braun, V., & Clarke, V. (2006). Using thematic analysis in psychology. *Qualitative Research in Psychology, 3*(2), 77–101. https://doi.org/10.1191/1478088706qp063oa
22. Glaser, B. G. (1965). The constant comparative method of qualitative analysis. Social problems, 12(4), 436-445.
23. Guest, G., Bunce, A., & Johnson, L. (2006). How many interviews are enough? An experiment with data saturation and variability. Field methods, 18(1), 59-82.
24. Marshall et al (2015). The HELPER programme: HEalthy Living and Prevention of Early Relapse – three exploratory randomised controlled trials of phase-specific interventions in first-episode psychosis. Appendix 5. The InterACT trial acceptability interview topic guide. https://www.ncbi.nlm.nih.gov/books/NBK285595/
25. Burke L, Littlewood E, Gascoyne S, McMillan D, Chew-Graham CA, Bailey D, et al. (2022) Behavioural Activation for Social IsoLation (BASIL+) trial (Behavioural activation to mitigate depression and loneliness among older people with long-term conditions): Protocol for a fully-powered pragmatic randomised controlled trial. PLoS ONE 17(3).
26. Irvine A, Drew P, Bower P, et al. Are there interactional differences between telephone and face-to-face psychological therapy? A systematic review of comparative studies. *Journal of Affective Disorders* 2020;265:120-31. doi: https://doi.org/10.1016/j.jad.2020.01.057
27. Hudson JL, Bower P, Kontopantelis E, et al. Impact of telephone delivered case-management on the effectiveness of collaborative care for depression and anti-depressant use: A systematic review and meta-regression. *PLOS ONE* 2019;14(6):e0217948. doi: 10.1371/journal.pone.0217948
28. Holmes, EA., O'Connor, RC., Perry, VH. et al. (2020) Multidisciplinary research priorities for the COVID-19 pandemic: a call for action for mental health science. Lancet Psychiatry. 7: p547-560.

For peer review only

For peer review only - http://bmjopen.bmj.com/site/about/guidelines.xhtml

Appendix 1 Themes from thematic analysis (TA)

Theme	Descriptive summary of theme	Illustrative data extracts
Intervention context	The COVID-19 restrictions applied variably to older adults in the BASIL study (e.g., shielding, social distancing) dependent on their health status. There was considerable variability in how the restrictions had impacted participants' mental health.	*I feel as though I'm held in, I'm being persecuted because I was shielding originally. I did everything that was asked of me, we're now moving out but all of a sudden because of political decisions, I'm now finding myself back where I was. Staying in a lot, don't meet a lot of people at the best of times, generally speaking you'll probably say loneliness, isolation. (OA06)*<br><br>*But I just didn't feel motivated to do anything 'cause all the things that I really had been enjoying had just suddenly stopped overnight, literally*. (OA14)<br><br>*It's just a worry, obviously I don't want to catch it, you know, because of my underlying conditions I think I would have a rough time of it, you know …So, I'm quite happy to isolate myself, if you like (OA12)*<br><br>*The constant changes in rules and the confusion that people have had over that, has, you know…it has affected it [intervention delivery]. Because one week, someone can be doing okay and the next week, rules have changed and they're feeling anxious, so they're not really sure where they stand anymore. Or, you know, restrictions have been lifted. And so one week, they're really struggling and the next week they've been able to*

		go back to doing most of what they normally do, and actually, they're loads better. So it changes week to week. So you really don't know from one week to the next, where someone's going to be at, dependent on, you know, the restrictions in their area. (BSW07)
	Remote consulting / perceived lack of access to primary care	*My diabetes, I think, they've forgot about me, I have no idea I haven't…the last two appointments were supposed to be phone ones and they didn't mat…they were cancelled for different reasons. My cancer ones, I don't know what's happening there, I've missed the last two because of the same problem. They've rang up to say that either the person's not available or the computer's broke down, or…so that's my cancer and my diabetes. (OA11)*<br><br>*There's been these two sides of things. There's one side, it's not affected their long-term conditions or their access in the support. The other side is where they're not wanting to access the support because they're either too scared or they don't want to go out, and then that's having the impact on their long-term conditions 'cause they're not being able to go to their routine appointments for it and things like that. (BSW06)*
Study entry	Phased approach to recruitment (i.e.: 1) initial text from GP surgery, 2) letter with study participant information (sent via GP surgery), 3) phone call from the Clinical Research	*So, I was really quite down in the dumps a bit. And then I got this text from my doctor saying that I should take part in…[they] thought it would be beneficial for me to take part. So, I said yes, I would do that and I rang the number and said yes I would.* (OA01)

For peer review only

	Network and 4) phone call from the University study team), was both feasible and acceptable. It seemed to provide successively layered opportunities for participants to gain sufficient information and decide if they wanted to participate in the BASIL study.<br><br>Need for targeted recruitment.	*No, I mean the doctor gives you a warning that this is about to happen and then you're prepared when somebody phones up that it's not a scam, a con, which is what I don't worry about it because I know how to deal with it but to some people it could be worrying. (OANC17)*<br><br>*I think it's maybe thinking about who we would want to target in the main study. I think as I say, the participants I was working with didn't consider themselves to be struggling with their mood, they'd come into the study for altruistic reasons, rather than looking to boost their own mood and wellbeing. I guess maybe to be more targeted. (BSW04)*
Intervention delivery and content	The BASIL pilot intervention was solely delivered by telephone to older adults in their homes. Online intervention delivery was offered; however, this option was not taken up by any participants.<br><br>Duration of intervention	*I: receiving the support package over the telephone, how was that?*<br>*P: Well, I found that the person I was speaking with showed a lot of empathy, understood where I was coming from, you know, just about being on your own for long periods of time and that sort of thing. (OA06)*<br><br>*And I thought, I don't want to be bothered with not being able to get it [online platform] on, you know, I'd rather a telephone call and then I don't have the worry of the [online platform] going wrong. (OA15)*<br><br>*So if they know they've got a telephone call on a*

For peer review only

		Wednesday morning at 11, then that will really help them to structure their week and give them something to look forward to and mark their weeks. (BSW05)<br><br>*So for those who have more severe low mood, when I start working with them, I definitely see that they have more to learn in terms of using technology. (BSW06)*
	Self-help booklet	*I think it was enough because we'd done the whole book and it left me, at the end, to have an action plan. So that's all there, I'd written out on the page, so I've got that to refer to. Yes, I think eight weeks was enough. (OA15)*
	BA	*My first thought was, it was in very simple, precise language so that anybody could understand it. It wasn't, you know, sort of above anybody's head if you know what I mean. I thought it was very good, very simple. And it had different examples of what people were doing and how they were overcoming their difficulties. Yes, I thought it was very good, I liked it. (OA15)*<br><br>*And there was also plenty of opportunity for me to put my two pennorth worth in, you had pages that were blank and…list your three most priority items today……. You know, that was so…so it gave you that. (OA06)*

For peer review only

		with the booklet and the [person] who I was talking with, we were able to say what is it about you that's so important that you must have this priority and that priority, and they compete, why don't you break it down. What can I do, how much can I do, how does it interwork with each other, and it's that breaking down of… catastrophic how you are, obviously, the pandemic, how does the pandemic impact you, what can you do to circumvent it, you know, that sort of thing. And that was very useful. (OA06)<br><br>*Also, probably the breaking things down into making them more manageable. That's been particularly useful for physical health. So that helped with the physical pain. (BSW06)*<br><br>*And I felt really good to tell {them] that it made me feel better, it made me feel good. The support I got from [them] was really good because things that I'd never thought about, [they] said [themselves], why don't you…you know, have you tried this? (OA01)*

For peer review only

Table 1

Consolidated criteria for reporting qualitative studies (COREQ): 32-item checklist

Number	Item	Guide questions/description	Location in text
Domain 1: Research team and reflexivity			
Personal Characteristics			
1.	Interviewer/facilitator	Which author/s conducted the interview or focus group?<br>Claire Sloan	Line 145, page 5
2.	Credentials	What were the researcher's credentials? E.g. PhD, MD<br>PHD in Psychology (University of Leeds)	Line 145, page 5
3.	Occupation	What was their occupation at the time of the study?<br>Research Fellow	Line 145, page 5
4.	Gender	Was the researcher male or female?<br>Female	Line 145, page 5
5.	Experience and training	What experience or training did the researcher have?<br>CS has extensive experience of the typical processes involved in health research, such as gaining REC and HRA approval, designing research protocols and recruiting/ interviewing vulnerable adults. She has also facilitated focus groups, PPI meetings, and analysed both qualitative and quantitative data for both her own research and studies she has assisted with. CS has the additional experience of thesis supervision and facilitating qualitative research seminars, as well as Deaf Awareness Training.	Line 148, page 5
Relationship with participants			
6.	Relationship established	Was a relationship established prior to study commencement?<br>No, this was avoided to eliminate possible bias	Line 145-146, page 5
7.	Participant knowledge of the interviewer	What did the participants know about the researcher? e.g. personal goals, reasons for doing the research<br>Participants had little knowledge about the interviewer (CS), just that she was researcher on the study interested in hearing about their experience of the intervention	Line 146, page 5
8.	Interviewer characteristics	What characteristics were reported about the interviewer/facilitator? e.g. Bias, assumptions, reasons and interests in the research topic.<br>N/A	
Domain 2: study design			
Theoretical framework			
9.	Methodological orientation and Theory	What methodological orientation was stated to underpin the study? e.g. grounded theory, discourse analysis, ethnography, phenomenology, content analysis<br>This research was based on two methodological approaches; the first was Thematic Analysis (TA) described by Braun and Clarke (2006) and the second was the Framework Method of Analysis, Gale et. al (2013), and its interpretation by Sekhon et.al (2017)	Line 165, page 5

For peer review only

Participant selection			
10.	Sampling	How were participants selected?<br>Consecutive sampling	Line 137, page 5
11.	Method of approach	How were participants approached? e.g. face-to-face, telephone, mail, email<br>Intervention participants who consented to being invited to a qualitative interview were contacted by telephone in order to check they were still happy to take part, and to arrange the interview. Basil support workers who provided their consent to be interviewed at their training were emailed to arrange the interview.	Line 143, page 5
12.	Sample size	How many participants were in the study?<br>15 intervention participants who completed the intervention (completers), 1 participant who did not complete the intervention (non completers) and 9 BSW's were involved in this qualitative research	Line 207-214, page 7
13.	Non-participation	How many people refused to participate or dropped out? Reasons? This is reported elsewhere and cited in the text	Line 210, page 7
Setting			
14.	Setting of data collection	Where was the data collected? e.g. home, clinic, workplace<br>Interviews were conducted remotely over the telephone.	Line 155, page 5
15.	Presence of non-participants	Was anyone else present besides the participants and researchers? No, these were one-to-one interviews (its possible that some interviewees had family members present but this was difficult to assess via telephone)	Line 155, page 5
16.	Description of sample	What are the important characteristics of the sample? e.g. demographic data, date<br>Demographic data is provided in the pilot trial paper, this is stated in the text.	Line 210, page 7
Data collection			
17.	Interview guide	Were questions, prompts, guides provided by the authors? Was it pilot tested?<br>Not provided. Pilot tested with individual from patient advisory group.	Line 203, page 7<br>Line 200-201, page 7
18.	Repeat interviews	Were repeat interviews carried out? If yes, how many?<br>No	Line 217, page 7
19.	Audio/visual recording	Did the research use audio or visual recording to collect the data?<br>Yes, an encrypted dictaphone was used to collect data from telephone interviews	Line 162, page 5
20.	Field notes	Were field notes made during and/or after the interview or focus group?	Line 164, page 5
21.	Duration	What was the duration of the interviews or focus group?	Line 163-164, page 5
22.	Data saturation	Was data saturation discussed?<br>Yes - we planned to recruit no more than 15 intervention completers as we anticipated we would hit data saturation at this point. During analysis it did become apparent that this was the case especially with particular domains of the TFA such as Burden (widely reported to be low burden to intervention participants) and Opportunity costs (widely reported to not require any sacrifice on behalf of the participants).	Line 212, page 7
23.	Transcripts returned	Were transcripts returned to participants for comment and/or correction?	Line 165, page 5

For peer review only

Domain 3: analysis and findings			
Data analysis			
24.	Number of data coders	How many data coders coded the data?	Line 192-197, page 7
25.	Description of the coding tree	Did authors provide a description of the coding tree?	N/A
26.	Derivation of themes	Were themes identified in advance or derived from the data?<br>Derived from the data	Line 220, page 7
27.	Software	What software, if applicable, was used to manage the data?<br>NVivo	Line 189, page 6
28.	Participant checking	Did participants provide feedback on the findings? No	Line 197-198, page 7
Reporting			
29.	Quotations presented	Were participant quotations presented to illustrate the themes / findings? Was each quotation identified? e.g. participant number<br>Yes	Line 234 - 487
30.	Data and findings consistent	Yes Was there consistency between the data presented and the findings?	N/A
31.	Clarity of major themes	Were major themes clearly presented in the findings?	N/A
32.	Clarity of minor themes	Is there a description of diverse cases or discussion of minor themes?	N/A

For peer review only
